# Mesenchymal precursor cells maintain the differentiation and proliferation potentials of breast epithelial cells

**DOI:** 10.1186/bcr3673

**Published:** 2014-06-10

**Authors:** Stephan Duss, Heike Brinkhaus, Adrian Britschgi, Erik Cabuy, Daniel M Frey, Dirk J Schaefer, Mohamed Bentires-Alj

**Affiliations:** 1 Friedrich Miescher Institute for Biomedical Research, Mechanisms of Cancer, Maulbeerstrasse 66, CH-4058 Basel, Switzerland; 2 Department of Surgery, University Hospital of Basel, Spitalstrasse 21, CH-4058 Basel, Switzerland

## Abstract

**Introduction:**

Stromal-epithelial interactions play a fundamental role in tissue homeostasis, controlling cell proliferation and differentiation. Not surprisingly, aberrant stromal-epithelial interactions contribute to malignancies. Studies of the cellular and molecular mechanisms underlying these interactions require *ex vivo* experimental model systems that recapitulate the complexity of human tissue without compromising the differentiation and proliferation potentials of human primary cells.

**Methods:**

We isolated and characterized human breast epithelial and mesenchymal precursors from reduction mammoplasty tissue and tagged them with lentiviral vectors. We assembled heterotypic co-cultures and compared mesenchymal and epithelial cells to cells in corresponding monocultures by analyzing growth, differentiation potentials, and gene expression profiles.

**Results:**

We show that heterotypic culture of non-immortalized human primary breast epithelial and mesenchymal precursors maintains their proliferation and differentiation potentials and constrains their growth. We further describe the gene expression profiles of stromal and epithelial cells in co-cultures and monocultures and show increased expression of the tumor growth factor beta (TGFβ) family member inhibin beta A *(INHBA)* in mesenchymal cells grown as co-cultures compared with monocultures. Notably, overexpression of INHBA in mesenchymal cells increases colony formation potential of epithelial cells, suggesting that it contributes to the dynamic reciprocity between breast mesenchymal and epithelial cells.

**Conclusions:**

The described heterotypic co-culture system will prove useful for further characterization of the molecular mechanisms mediating interactions between human normal or neoplastic breast epithelial cells and the stroma, and will provide a framework to test the relevance of the ever-increasing number of oncogenomic alterations identified in human breast cancer.

## Introduction

Breast cancer is a progressive and heterogeneous disease that arises in the epithelial cells of glands. Factors contributing to the progression and heterogeneity of breast cancer include the differentiation state of the cancer cell of origin, the number and nature of the transforming events, and microenvironmental cues [1-5]. In the presence of the same transforming events, the differentiation state of the cells used for modeling breast cancer may still influence the tumorigenicity, histology and metastatic potential of the resulting tumors [1]. Hence, it is essential to consider the cell hierarchy of the human breast and to control the differentiation states of cells used to model and study breast cancer.

The breast epithelium is embedded in stromal tissue consisting of extracellular matrix (ECM), mesenchymal, endothelial and immune cells. The epithelium of the mouse and human mammary gland is organized hierarchically and encompasses undifferentiated stem/progenitor cells and differentiated luminal epithelial and basal myoepithelial cells [6-11]. Stemness is a dynamic property tightly controlled by the stem cell niche, which is a dedicated microenvironment supposedly containing specialized stromal and epithelial cell types as well as a defined ECM [12-16]. The niche regulates tissue homeostasis by controlling mammary stem cell quiescence and activation under the influence of systemic and local cues [17-19].

In mice, bone marrow mesenchymal stem cells (MSCs) and hematopoietic stem cells were shown to interact and form a bone marrow niche [20]. MSCs were discovered originally in the bone marrow but later described in many tissues [21,22]. They display a vast differentiation potential, giving rise to mesodermal and non-mesodermal cell lineages such as osteocytes, adipocytes, chondrocytes, myocytes, cardiomyocytes, fibroblasts, myofibroblasts, endothelial cells, and neurons [23-25]. Epithelial-mesenchymal interaction has been shown to contribute to mouse mammary tissue homeostasis [26-29]. In humans, the results of morphological studies of embryos have suggested a role for epithelial-mesenchymal interactions in breast development [30]. Whether MSCs and/or their progeny contribute to the maintenance of human undifferentiated breast cells remains unknown.

Gene expression profiling and genome-wide sequencing of human breast tumors has revealed a multitude of alterations [31-33]. There is an urgent need for physiologically relevant *ex vivo* culture systems based on primary breast cells in which the significance of identified alterations can be tested. Such culture systems should recapitulate features of human breast such as cellular composition and differentiation states [34,35].

Here we describe *ex vivo* cell culture conditions that allow the maintenance and propagation of human breast-derived primary epithelial and mesenchymal cells simultaneously. Co-culture of primary mesenchymal and epithelial precursor cells on coated meshes allows long-term maintenance of the differentiation potentials of human breast epithelial and mesenchymal precursors. Moreover, breast mesenchymal precursor cells constrain the growth and spreading of primary epithelial cells in heterotypic cultures. We further describe the gene expression profiles of stromal and epithelial cells in co-cultures and monocultures and show that mesenchymal inhibin beta A *(INHBA)* increases the colony formation potential of breast epithelial cells.

## Material and methods

### Cell culture

Reduction mammoplasty tissue was obtained with appropriate informed consent from the patients and fresh samples used to isolate primary human breast epithelial cells (PHBECs) and human adipose tissue-derived mesenchymal stem cells (hAMSCs) using modifications of published protocols [36-39]. Approval for the study was granted by the *Ethic Commission Beider Basel* (EKBB). In brief, adipose tissue was separated from epithelial breast tissue by gentle scraping with sterile scalpels. Tissue that contained fine epithelial filaments or strongly vascularized tissue was discarded and only yellow tissue was processed further. The selected tissue was washed twice with equal volumes of PBS and 100-μm cell strainers (BD, Falcon) were used to drain PBS. The tissue was chopped with sterile scalpels until a homogenous, almost liquid mass was obtained. The fat tissue was digested for 2 h with digestion medium comprising DMEM/F12, 15 mM 4-(2-hydroxyethyl)-1-piperazineethanesulfonic acid (HEPES), 1 × penicillin/streptomycin, insulin (10 μg/mL), hydrocortisone (0.5 μg/mL), and collagenase type A from *Clostridium histolyticum* (Roche Diagnostics) (1 mg/mL, >0.15 U/mg) at 37°C on a rotating wheel. The solution was centrifuged for 5 minutes at 192 *g* and the supernatant discarded. Resulting pellets containing erythrocytes were incubated for 10 minutes in red blood cell lysis buffer. The solution was then filtered with 70-μm cell strainers (BD, Falcon) in new tubes and equal volumes of MSCM (DMEM/F12 supplemented with 15 mM HEPES, 10% fetal calf serum, 1 nM 17-β-estradiol, and 1 × penicillin/streptomycin). Cells were then centrifuged for 5 minutes at 192 *g* and washed twice with MSCM before re-suspension in MSCM and filtering through 40-μm cell strainers (BD, Falcon) to remove large debris. Cells were counted, plated at densities of 500 to 1,000 cells per 10 cm^2^ and incubated at 37°C in a humidified 5% CO_2_/O_2_ controlled incubator. After 24 h, media was removed, the cells washed with PBS, and fresh medium added to expand cells for 14 days before freezing. Breast epithelial cells were cultured in mammary epithelial cell growth medium (MEGM) [40], human mammosphere medium (HMM) [41] or M5 medium comprising 50% M199 medium (ANIMED/Bioconcept), 50% F12 (SIGMA) supplemented with 20 ng/mL epidermal growth factor (EGF) (PeproTech DE), 1 × B-27 (Invitrogen/GIBCO), 1 nM 17-β-estradiol, 57 μM β-mercaptoethanol, 15 mM Hepes (SIGMA) and 1 × penicillin/streptomycin (Invitrogen/GIBCO). M5 was used for all experiments unless stated otherwise. Uncoated tissue culture plastic (BD, Falcon Primaria) was used for monolayer colony formation and differentiation assays of PHBECs. From 500 to 2,000 cells were seeded per well in 6-well plates and grown in M5 medium for 7 to 10 days prior to fixation. A published method [40] was adapted to test the potential for differentiation into β-casein-secreting alveolar cell lineages (lactogenesis assay). Briefly, PHBECs were seeded as for the colony formation assay and grown in M5 medium for 10 days. The colonies were then overlaid with matrigel and incubated for 2 days before addition of the differentiation medium (M5 medium supplemented with hydrocortisone 1 μg/mL, insulin 5 μg/mL, and recombinant human prolactin 1 μg/mL, all from SIGMA). Half of the medium was replaced by fresh differentiation medium every second day and cells were fixed 7 days after exposure to differentiation medium. All cells were cultured at 37°C in a humidified 5% CO_2_, 5% O_2_ controlled incubator.

### Lentivirus infections

CFP from pECFP (Clontech) and VENUS NLS [42] were cloned into pRRLhPGK.GFP.SIN18 [43] using BamHI and BsrGI restriction sites and lentiviruses were produced by calcium phosphate transfection of 293 T cells as described [43]. The titer of each lentiviral batch was determined on PHBECs. hAMSCs grown on Primaria plates were infected for 6 h in the first passage in the presence of hexamethrine bromide (8 μg/mL, SIGMA) and the cells were selected 48 h later with 0.8 μg/mL puromycin (Invitrogen) for 5 days. Freshly dissociated PHBECs were infected in suspension for 6 h in the presence of hexamethrine bromide (8 μg/mL) and then grown in ultra-low attachment (ULA) dishes (Corning) for 6 days. All infections were performed at a multiplicity of infection of 20 viral particles per cell. Selection with 0.8 μg/ml puromycin was applied 48 h after infection. The spheres formed were dissociated and the resulting single cells aggregated.

### *Ex vivo* niche cultures

Cell strainers (40 μm; BD, Falcon) were coated with a mixture of 100 μg/mL rat tail collagen (Roche) and 10% (v/v) Matrigel (BD). Rat tail collagen (Roche) was reconstituted with sterile 0.2% acetic acid (v/v) for at least 6 h at 4°C and then diluted to obtain a final concentration of 100 μg/ml collagen in M199/F12 (50% M199 (ANIMED/Bioconcept), 50% F12 (SIGMA) plus 15 mM HEPES (Invitrogen/GIBCO)). The mixture was briefly vortexed and chilled on ice for 10 minutes. Matrigel (10% v/v) (BD) was added and the mixture vortexed for 10 s and chilled on ice for 10 minutes. In the meantime, the noses of 40-μm cell strainers were cut with sterile curved surgical tissue scissors and the strainers placed in 6-well tissue culture plates (Corning). Collagen/Matrigel mixture (1.5 ml) was slowly and carefully added to the center on top of the nylon mesh. The strainers were incubated at 37°C for 30 minutes before being removed from the plates and turned upside down to discard spare collagen/Matrigel mixture. The coated strainers were then carefully transferred to fresh 6-well plates for washing with 3 mL of pre-warmed (37°C) M199/F12. After this washing step, the coated strainers were transferred into ULA 6-well plates (Corning) containing 3 mL of pre-warmed (37°C) M5 medium. The plates were incubated at 37°C in a humidified 5% CO2, 5% O2 controlled incubator until cell aggregates were added. Equal numbers of hAMSCs and PHBECs were aggregated at densities of 100,000 cells per mL of M5 medium in ULA dishes. After 4 to 6 days, the aggregates were collected by mild centrifugation (50 *g* for 30 s) and seeded on coated 40-μm cell strainers; 25% (v/v) of fresh medium was added every 2 to 3 days and replaced completely every 8 to 9 days.

### Dissociation of *ex vivo* niche cultures

Cells were dissociated with HyQtase (HyClone, Thermo Scientific) for 20 minutes on a rotating wheel at 37°C. Subsequent pipetting for 5 minutes and filtering through 40-μm cell strainers (Falcon) yielded single cells that could be used for cell sorting, colony formation and differentiation assays.

### Antibodies and immunochemistry

Only cells without fluorescent protein tags were stained. Cells were fixed with ice-cold methanol/acetone (50/50 v/v) for 10 minutes at room temperature. The cells were incubated at 4°C overnight with the following primary antibodies: keratin 14 (RB-9020, 1:4,000), keratin 18 (MS-142, 1:2,000), keratin 19 (MS-198 1:1,000), β-casein (MS-935 1:1,000), Ki67 (RB-1510, 1:500) (Thermo Scientific, Stehelin), p63 (MS-1081, 1:200) (Thermo Scientific), e-cadherin (610181, 36/Ecad, 1:1,000) (BD Bioscience,), EpCAM (Clone 9C4, 1:100) (BioLegend), or keratin 8/18 (GP11, 1:500) (Fitzgerald). Goat anti-mouse, goat anti-rabbit or goat anti-guinea pig secondary antibodies coupled to Alexa 488, 568 or 633 (Molecular Probes, Invitrogen 1:500) were used for detection.

### Microscopy and image analysis

For phase-contrast and fluorescence imaging of adherent cells and floating *ex vivo* cultures, images were acquired with an inverted microscope (Nikon Eclipse Ti) using a 10× lens (Nikon Plan Fluor, NA 0.3) and a Nikon Ds-Fi camera. Immunofluorescent staining was analyzed with an inverted Zeiss Z1 microscope using a 20× air lens (Zeiss Plan-APOCHROME, NA 0.8) equipped with a motorized Zeiss scanning stage. Axiovision software was used to acquire and stitch images. For confocal microscopy, meshes were mounted between glass slides and coverslips with ProLong anti-fade reagent (Invitrogen) before analysis on an LSM-510 confocal microscope (Zeiss) using a 20× air lens (Zeiss Plan-APOCHROME, NA 0.8). Both Zeiss microscopes were equipped with an Axio Cam MRC CCD (6.45 micron). The Image-J (Fiji (64 bit)) software [44] was used for image quantifications and three-dimensional reconstruction.

### hAMSCs differentiation assays

Approximately 50,000 first-passage hAMSCs from reduction mammosplasties were seeded in Primaria 6-well dishes and cultured in MSCM overnight. The medium was then substituted with MSCM or M5 medium containing 5 μg/mL insulin, 1 μM dexamethasone, 0.5 μM isobutylmethylxanthine, 60 μM indomethacin and 10 nM 17-β-estradiol (SIGMA) for adipogenic differentiation, or with 1 μM dexamethasone, 10 mM β-glycerophosphate, 100 μM ascorbic acid 2-phosphate, and 10 nM 17-β-estradiol for osteogenic differentiation. The cultures were maintained for 20 days before being fixed with formalin. Adipogenic differentiation was determined by staining with Oil red O (0.3%; SIGMA) in isopropanol (57%) and water to detect lipid. Staining of alkaline phosphatase activity (SIGMAFAST BCIP, SIGMA) was carried out to assess osteogenic differentiation.

### Flow cytometry

For sorting of fluorescent cells from *ex vivo* cultures, the cells were filtered with 40-μm cell strainers (Falcon) after dissociation: 4',6-diamidino-2-phenylindole (DAPI) (0.2%, Invitrogen) was added (1:250) 2 minutes before cell sorting. Fluorescence-activated cell sorting (FACS) was performed with a MoFlo cell sorter (Becton Dickinson). Single cells were gated based on their forward and side scatter profiles; dead cells (DAPI bright) were gated out. For sorting of cells from normal breast tissue, organoids were dissociated with HyQtase (HyClone, Thermo Scientific) for 10 minutes at 37°C and subsequent pipetting. Cells were filtered twice through 40-μm cell strainers (Falcon) to obtain single cells; 10^6^ cells were blocked in M5 medium for 10 minutes at 4°C with antibodies against human CD16 (FcRIII, Clone 3G8, 1:50) and CD32 (FcRII, Clone FUN-2, 1:100), then washed and labeled in 100 μl M5 medium for 20 minutes at 4°C with antibodies against human FITC-CD49f (Clone GoH3, 1:25), PerCP/Cy5.5-CD326 (EpCAM, Clone 9C4, 1:25), APC-CD10 (Clone HI10a, 1:20), PE-CD31 (Clone WM59, 1:33), PE-CD45 (Clone HI30, 1:33), and PE-CD235ab (Clone HIR2, 1:33). DAPI (0.2%, Invitrogen) was added (1:250) 2 minutes before cell sorting. Single cells were gated based on their forward and side scatter profiles. Dead cells (DAPI bright) and Lin + cells (CD31+, CD45+ and CD235+) were gated out (see Additional file 1). All antibodies were purchased from BioLegend. The cells were directly sorted into TRIzol (Invitrogen).

### Microarray profiling

Viable cells were FACS-sorted into TRIzol (Invitrogen). RNA was extracted from 300 sorted cells with the PicoPure RNA isolation kit (Arcturus) and reverse-transcribed using 4 μM T7- (dT)24/T7-(dN)6 primer mix (Affymetrix) and 150 units of Superscript II reverse transcriptase (Invitrogen). The synthesis of second-strand cDNA was performed by mixing 4 mM dNTPs, 6 units DNA polymerase I, and 0.4 units RNase H in a 20-μL reaction volume. cRNA was produced by *in vitro* transcription with a T7 RNA polymerase at 37°C for 14 h using the MEGAscript T7 kit (Ambion) as per the manufacturer's instructions. For the second cycle, the first-strand cDNA was synthesized using 0.2 μg random primers from 9 μL of purified cRNA. The second-strand cDNA was produced using 10 μM T7- (dN)6 primer and 40 units DNA polymerase at 16°C for 2 h, after which 10 units of T4 DNA polymerase (Invitrogen) was added and the incubation continued for another 10 minutes. The cDNA was *in vitro* transcribed with T7 RNA polymerase at 37°C for 16 h. The single-strand cDNA was synthesized using 10 μg purified cRNA in the presence of 4 μg random primers, 0.2 M DTT, 12 mM dNTP + dUTP, and 750 units Superscript II (Roche Diagnostics) in a total volume of 20 μL. The cRNA was hydrolyzed with 2 units RNase H at 37°C for 40 minutes. The sense cDNA was purified and eluted in 28 μL elution buffer. Amplified products were purified using the GeneChip cDNA Sample Cleanup Module (Affymetrix) with a 6,000-*g* centrifugation during the first two steps. To improve recovery from the columns, water or elution buffer was spun into the matrix at 50 *g* and left to stand for 4 minutes before a 16,000-*g* centrifugation. The quantity and purity of the cRNA and cDNA produced during the first and second rounds were evaluated using a NanoDrop ND-1000 spectrophotometer (Nanodrop Technologies). The cDNA was then fragmented by uracil DNA glycosylase and apurinic/apyrimidic endonuclease 1 and biotin-labeled with terminal deoxynucleotidyl transferase using the GeneChip WT Terminal labeling kit (Affymetrix). Following hybridization, non-specifically bound nucleotides were removed by washing and specifically bound target detected using a GeneChip Hybridization, Wash and Stain kit and a GeneChip Fluidics Station 450 (Affymetrix). Hybridization was carried out with 5 μg of biotinylated target, which was incubated with a GeneChip human Gene 1.0 ST Array (Affymetrix) at 45°C for 16 h. The arrays were scanned using a GeneChip Scanner 3000 7G (Affymetrix) and CEL files acquired using GeneChip Command Console Software (Affymetrix). Arrays were normalized and probeset-level expression values calculated with R/Bioconductor's (v2.14) *affy* package using the rma() function [45]. Differential gene expression was determined using linear modeling implemented in the R/Bioconductor package *limma*. For general analysis, a cutoff of linear fold change >2 and *P*-value <0.005 were used. Lists of differential genes were imported into Ingenuity IPA (Ingenuity Systems, [46], content version 12710793) for pathway analysis. Gene set enrichment analysis (GSEA) was performed using the JAVA application from the Broad Institute v2.0 [47]. GSEA and gene ontology data mining (ontologizer [48]) were performed with the gene lists discussed. The microarray data from this publication have been submitted to the NCBI’s Gene Expression Omnibus [49] and are accessible (see [50,51]).

### Immunoblotting

Cells were lysed with radioimmunoprecipitation assay (RIPA) buffer (50 mM Tris–HCl pH 8, 150 mM NaCl, 1% NP-40, 0.5% sodium deoxycholate, 0.1% SDS) supplemented with 1 × protease inhibitor cocktail (Complete Mini, Roche), 0.2 mM sodium orthovanadate, 20 mM sodium fluoride, and 1 mM phenylmethylsulfonyl fluoride (SIGMA); 25 μg of protein lysate was loaded onto 8% SDS PAGE gels. Immunoblots were performed with antibodies against ITG11A1 (Abnova, 1:700), COL11A1 (Abcam 1:1,000), MMP13 (Thermo Scientific, 1:500), INHBA (Abcam, 1:500), SULF1 (Abcam, 1:500), EPYC (SIGMA, 1:2,000), TNFSF4 (BioLegend, 1:300) and ERK2 (Santa Cruz Biotechnology, 1:2,000) overnight at 4°C.

### Statistical analysis

Data were tested for normal distribution and the standard two-tailed *t*-test or analysis of variance (ANOVA) was applied. The Tukey-Kramer honest significant difference (HSD) test was performed to account for multiple comparisons. Excel 2010 and JMP11 (SAS) were used for all statistical tests. Experiments were carried out with at least three biological replicates unless stated otherwise.

## Results

### Isolation and propagation of human breast epithelial and mesenchymal precursor cells

**Figure 1:**
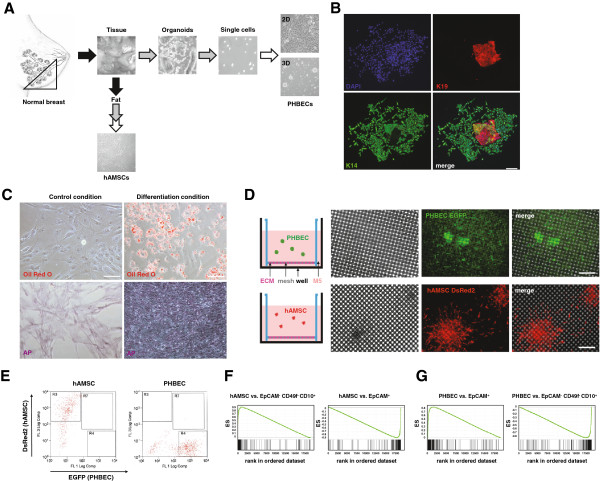
**Human breast mesenchymal and epithelial precursor cells maintain their identities in *ex vivo *cell culture conditions. (A)** Schematic of the experimental procedure for the isolation of mesenchymal stem cells human adipose tissue-derived mesenchymal stem cells (hAMSCs) and primary human breast epithelial cells (PHBECs) from normal breast reduction mammoplasty tissues. Arrows: mechanical (black) and enzymatic (gray) digestion steps. Isolated epithelial cells can be grown either as monolayer cultures (two-dimensional) or as three-dimensional cultures. **(B)** Colony formation assay with single cells derived from mammospheres grown in M5 medium. Representative images of K19 (red) and K14 (green) immunofluorescent staining of PHBEC colonies: 4',6-diamidino-2-phenylindole (DAPI)- (blue) stained nuclei. Scale bar 100 μm. **(C)** Differentiation assays of mesenchymal precursor cells in M5 medium. Representative images of adipocytes and osteocytes stained with lipid specific Oil Red O or alkaline phosphatase (AP), respectively. Scale bars 10 μm. **(D)** Schematics of *ex vivo* monoculture conditions of PHBEC (upper) and hAMSC (lower) with representative phase-contrast images (left) and fluorescence images of PHBECs expressing enhanced green fluorescent protein (EGFP) and hAMSCs expressing red fluorescent protein (DsRed2) after 30 days growth on an extracellular matrix (ECM)-coated mesh. Scale bars 200 μm. **(E)** Representative flow cytometry dot plots showing separated DsRed2-expressing mesenchymal cells (left) and EGFP-expressing epithelial cells (right) prior to RNA extraction and transcription profiling. **(F)** Gene set enrichment analysis (GSEA) with hAMSC-specific genes and signatures of EpCAM- CD49f- CD10+ and EpCAM + breast cells isolated from normal breast tissue. hAMSC/EpCAM- CD49f- CD10+: enrichment score (ES) = 0.87, normalized enrichment score (NES) = 3.2, false discovery rate (FDR) <0.0001, *P* <0.0001; hAMSC/EpCAM+: ES = −0.87, NES = −3.18, FDR <0.0001, *P* <0.0001. **(G)** GSEA with PHBEC-specific genes and signatures of EpCAM + and EpCAM- CD49f- CD10+ breast cells. PHBEC/EpCAM+: ES = 0.85, NES = 3.29, FDR <0.0001, *P* <0.0001; PHBEC/ EpCAM- CD49f- CD10+: ES = −0.85, NES = −3.32, FDR <0.0001, *P* <0.0001.

The human breast is composed of a network of ductal and alveolar epithelial cells embedded in a stroma that includes connective and fatty tissues. We isolated mesenchymal and epithelial breast precursor cells and defined cell culture conditions that allow the maintenance and the propagation of these precursors simultaneously and their differentiation upon exposure to specific cues. We adapted previously described experimental procedures for the isolation of PHBECs and hAMSCs from breast tissue [36,38]. Human breast reduction mammoplasty tissue samples were subjected to mechanical and enzymatic dissociation steps to obtain single-cell suspensions of epithelial and fat tissue fractions (Figure 1A). To enrich for epithelial stem/progenitor cells while depleting differentiated cells, we cultured the epithelial fraction in low-density suspension conditions on ULA plates as described previously [41]. Homogenous hAMSC cultures were obtained by seeding single cells from the breast adipose tissue fraction as low-density monolayer cultures.

As our ultimate goal was to co-culture mesenchymal and epithelial precursor cells, it was essential to define cell culture conditions that allow the simultaneous growth of both mesenchymal and epithelial precursors without compromising their differentiation potential. To this end, we developed a novel serum-free cell culture medium (M5) by combining the previously described HMM and WIT media [1,41]. We maintained cultures at 5% oxygen tension to reduce oxidative stress and to culture primary cells in physiological conditions [52,53]. For monolayer culture and/or differentiation assays, cells were grown on a tissue culture surface with negatively charged oxygen- and positively charged nitrogen-containing functional groups (BD Primaria), which aided the attachment and culture of primary cells [1,54,55].

We compared the differentiation potential of epithelial cells seeded at low densities and cultured for four passages (28 days) in M5, MEGM [40] or HMM [41] culture media (see Additional file 2A). The proportions of luminal epithelial and myoepithelial cells in these cultures were monitored by immunostaining for keratin 18 (K18) and keratin 14 (K14), respectively. Epithelial cells cultured in MEGM were mostly large, non-proliferating and K14-positive, with 3 ± 2.5% K18-positive cells. Cells grown in HMM were also mostly K14 positive with 6 ± 0.7% of K18-positive cells. In contrast, M5 medium allowed more than three- and six-fold enrichment of luminal cells compared with the HMM and MEGM culture media, respectively, with 19.8 ± 0.4% K18-positive cells (see Additional file 2A). Moreover, PHBECs cultured in M5 medium covered a larger area of the culture surface than cells grown in HMM, and were smaller than cells grown in either HMM or MEGM (see Additional file 2A, B). Thus, we successfully defined culture conditions that allow the differentiation and propagation of both luminal and myoepithelial breast cell lineages.

M5 medium was then tested for the enrichment of breast epithelial precursor cells. PHBECs grown in suspension in M5 medium formed mammospheres [40] at frequencies of 5.4 ± 0.9 spheres per 1,000 cells and thus exhibited mammosphere formation frequencies similar to breast epithelial cells grown in MEGM (5.9 ± 0.5 spheres per 1,000 cells) or HMM medium (5.3 ± 0.6 spheres per 100 cells) (see Additional file 2C). Mammospheres grown in M5 medium were then dissociated and their colony formation capacity, a surrogate readout for progenitor/stem cells [40], was assessed. Circa 1/200 single cells derived from mammospheres grown in M5 medium formed mixed colonies of luminal K19-positive and myoepithelial K14-positive mammary epithelial cells (Figure 1B). Notably, 2 to 8% of cells in the colonies were identified as K14 and K19 double-positive (yellow in merge Figure 1B), a characteristic of putative breast stem/progenitors [56]. Triple staining for K18, K14 and the myoepithelial marker p63 showed that 95.4% of K18-positive cells were negative for p63, whereas 93.4% of K14 positive cells also had nuclear p63, thus confirming their myoepithelial nature (see Additional file 2D). These data demonstrate that M5 medium allows the propagation of undifferentiated bi-potent epithelial precursor cells in suspension and the differentiation of such cells along the two breast epithelial lineages when seeded in colony formation assays on a tissue culture surface.

hAMSCs are usually maintained in serum-containing mesenchymal stem cell medium [57]. To address whether hAMSC can also be maintained and differentiated in serum-free M5 medium, we compared the differentiation potential of hAMSCs cultured in standard mesenchymal stem cell medium (MSCM) or M5 medium. Upon addition of adipogenic or osteogenic factors to MSCM (see Additional file 2E) or M5 (Figure 1C), hAMSCs differentiated into cells expressing adipocytes and osteocytes markers, respectively, as depicted by adipocyte differentiation-specific Oil red O and osteocyte differentiation-specific AP staining. These data show that isolated hAMSCs maintain their differentiation potential when grown in serum-free M5 medium.

Altogether the data demonstrate that our methods allow the isolation and culture of mesenchymal and epithelial precursor cells from human breast tissue, as well as the propagation and differentiation of breast epithelial and mesenchymal precursor cells.

### PHBECs and hAMSCs maintain their identity when grown on nylon meshes submerged in M5 medium

Immortalized breast epithelial cells grown in three-dimensional culture recapitulate features of the normal breast [58,59]. We cultured non-immortalized human primary breast cells for 3 weeks in monolayer, three-dimensional Matrigel, or ULA suspension conditions but they failed to grow after passaging. We then set out to define three-dimensional cell culture conditions that maintain the identities, differentiation and growth potential of human primary epithelial and mesenchymal cells for a long period. To be able to trace cells, we infected freshly isolated PHBECs or hAMSCs with lentiviral constructs bearing fluorescent proteins in suspension. PHBECs and hAMSCs were successfully tagged with nuclear enhanced yellow fluorescent protein (VenusNLS) or red fluorescent protein (DsRed2), respectively (see Additional file 2F). We then cultured tagged PHBECs or hAMSCs in suspension to allow them to form aggregates, which were then seeded onto nylon meshes coated with a mixture of collagen I and laminin-rich Matrigel (Figure 1D). The aggregates settled on the ECM-coated meshes and the cells invaded the substrate (Figure 1D). To assess whether the PHBECs and hAMSCs preserved their molecular identities when grown as monocultures on coated meshes after prolonged *ex vivo* culture, we dissociated them after 30 days *ex vivo* culture, used FACS to sort viable cells (Figure 1E), and analyzed the global transcriptome of these cells. Pearson correlation matrix of the arrays revealed distinct gene expression patterns for PHBECs and hAMSCs (see Additional file 3A) and unsupervised hierarchical clustering of expression data yielded clusters enriched in mesenchyme- and epithelia-specific genes, respectively. These data demonstrate the successful isolation, tagging and maintenance of primary mesenchymal and epithelial breast cells (see Additional file 3B).

To further validate the molecular identities of epithelial and mesenchymal breast cells after prolonged *ex vivo* culture, we set out to compare them to freshly isolated breast cells. Therefore, we sorted freshly dissociated cells from normal breast tissue organoids by FACS (see Additional file 1) and performed gene expression analysis of lineage-negative (Lin-) epithelial cell subpopulations (EpCAM + or EpCAM- CD49+) [2] as well as mesenchymal cell subpopulations (EpCAM- CD49f- CD10+ or EpCAM- CD49f- CD10-) [60]. We then compared the profiles by GSEA [61,62]. We observed a very significant overlap between the gene expression profiles of hAMSCs from prolonged *ex vivo* culture and the profiles of freshly isolated Lin- EpCAM- CD49f- CD10+ mesenchymal cells but not with EpCAM + epithelial cells (Figure 1F). In addition, we found very significant overlap between the gene expression profiles of PHBECs from prolonged *ex vivo* culture and the profiles of freshly isolated Lin- EpCAM + epithelial cells but not with EpCAM- CD49f- CD10+ mesenchymal cells (Figure 1G). These results further validate the isolation methods and *ex vivo* culture conditions maintaining the molecular identities of epithelial and mesenchymal breast cells.

We also compared the gene-expression profiles of PHBECs and hAMSCs (see Additional file 4A, B) to previously published profiles of dissociated breast cells [63,64] (see Additional file 4C, D) and to the primary breast epithelial cells (BPECs) and (HMECs) that were shown to display two differentiation states; with HMECs expressing two-fold more myoepithelia-specific genes than BPECs [1] (see Additional file 4E). Notably, GSEA revealed the gene expression signature of PHBECs cultured on coated meshes to be highly correlated with the signature of EpCAM + epithelial cells, luminal progenitor cells, and BPECs but not CD10+ myoepithelial/myofibroblast cells, basal cells or HMECs, respectively (see Additional file 4C-E) [1,63,64]. Furthermore, the gene expression signature of hAMSCs cultured in our *ex vivo* culture conditions, but not PHBECs, overlapped highly significantly with published MSC profiles [65,66] (see Additional file 4F, G). In summary, these data confirm that the *ex vivo* cell culture conditions described here maintain the epithelial and mesenchymal identities of PHBECs and hAMSCs, respectively.

### Breast epithelial and mesenchymal precursors constrain growth and spreading reciprocally in heterotypic cultures

**Figure 2:**
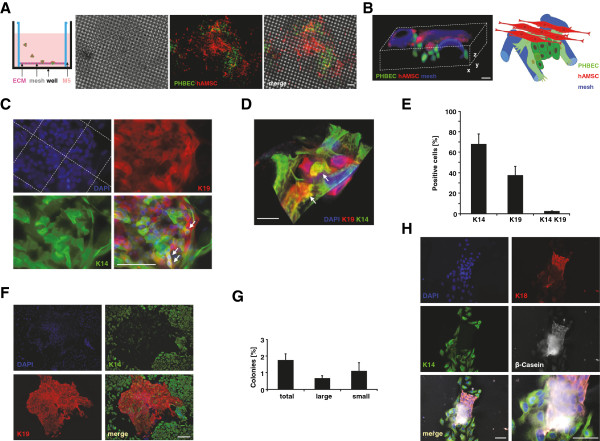
**Heterotypic three-dimensional cultures maintain tri-potent mammary epithelial precursor cells. (A)** Schematic of primary human breast epithelial cells (PHBECs) and human adipose tissue-derived mesenchymal stem cells (hAMSCs) co-culture, phase-contrast (left) and fluorescence images of clusters of the co-cultures grown for 30 days on an extracellular matrix (ECM)-coated mesh. Scale bar 100 μm. **(B)** Three-dimensional reconstruction of 28 z-sections (0.6 μm) from confocal images of a 30-day-old co-culture of tagged-PHBECs (VenusNLS, green) and hAMSCs (DsRed2, red) on ECM-coated mesh. Autofluorescence of nylon mesh (blue). Scale bar 10 μm. (**C**) Confocal images of K14- and K19-stained 60-day-old co-cultures of untagged cells: 4',6-diamidino-2-phenylindole (DAPI)- (blue) stained nuclei. Arrows indicate double-positive putative bi-potent epithelial progenitor cells. Scale bar 50 μm. **(D)** Three-dimensional reconstruction of 48 z-sections (0.4 μm) from confocal images of K14-, K19- and DAPI-stained co-cultures (cells without fluorescent protein tags were stained). Scale bar 10 μm. Arrows indicate double-positive cells. **(E)** Percentages of K14, K19 and K14/K19 double-positive cells in co-cultures. K14 versus K19, *P* = 0.10714; K19 versus K14K19, *P* = 0.00019; K14 versus K14K19, *P* = 0.01444. **(F)** Colony formation assay. Fluorescence images of K14- and K19-stained colonies formed by single cells derived from co-cultures of untagged PHBECs and hAMSCs. DAPI- (blue) stained nuclei. Scale bar 50 μm. **(G)** Percentage of colonies formed by single cells derived from co-cultures. Total: all colonies (n >8 cells); large: large mixed colonies (n >32 cells, >4 doublings); small: small colonies (n = 8 to 32 cells, 2 to 4 doublings). Small versus large, *P* = 0.46563; total versus large *P* = 0.13752; total versus small, *P* = 0.29473. **(H)** Lactogenesis assay. Fluorescence images of K14-, K18- and β-casein-stained colonies derived from single cells from co-cultures grown in lactogenic differentiation conditions. DAPI- (blue) stained nuclei. Lower right corner: 2.5-fold magnification of merged image. Scale bars 50 μm.

**Figure 3:**
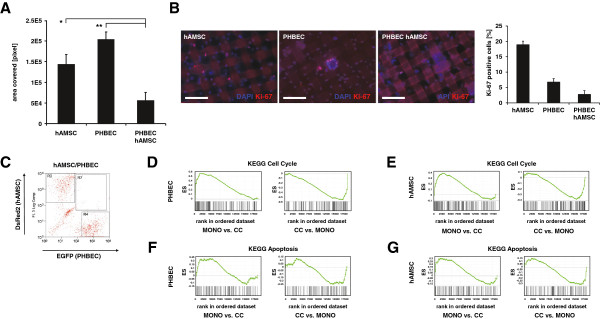
**Human adipose tissue-derived mesenchymal stem cells (hAMSCs) and primary human breast epithelial cells (PHBECs) in co-culture restrain their spreading and their proliferation reciprocally. (A)** Bar graph showing the area of the mesh covered by monocultures of fluorescent protein-tagged hAMSCs or PHBECs, and of hAMSC/PHBEC co-cultures. Results represent means ± SD from three independent experiments; *P <0.01, **P <0.001. **(B)** Fluorescent images of Ki67 immunostained *ex vivo* cultures (red): 4',6-diamidino-2-phenylindole (DAPI) (blue) shows nuclei. Bar graph shows percent of Ki67-positive cells detected in each culture condition. Cells without fluorescent protein tags were stained. Scale bars 100 μm. hAMSC versus co-culture (CC), *P* = 7.39E-05; PHBEC versus CC, *P* = 0.01312; hAMSC versus PHBEC, *P* = 0.00018. **(C)** Representative flow cytometry dot plot showing separated red fluorescent protein (DsRed2)-expressing mesenchymal cells and enhanced green fluorescent protein (EGFP)-expressing epithelial cells from *ex vivo* co-culture prior to RNA extraction and transcription profiling. **(D)** Gene set enrichment analysis (GSEA) with PHBEC-specific genes and KEGG signatures comprising cell cycle genes (MSigDB), GSEA scores: mono/CC enrichment score (ES) = 0.58 normalized enrichment score (NES) = 2.38, CC/Mono ES = −0.58 NES = −2.38, false discovery rate (FDR) <0.0001, *P* <0.0001. **(E)** GSEA with hAMSC-specific genes and KEGG signatures comprising cell cycle genes (MSigDB), GSEA scores: mono/CC ES = 0.41, NES = 1.6, CC/Mono ES = −0.41, NES = −1.6, FDR = 0.0082, *P* = 0.005. **(F)** GSEA with PHBEC-specific genes and KEGG signatures comprising apoptosis genes (MSigDB), GSEA scores: mono/CC ES = 0.25, NES = 0.98, CC/Mono ES = −0.25, NES = −0.98, FDR = 0.866, *P* = 0.476. **(G)** GSEA with hAMSC-specific genes and KEGG signatures comprising apoptosis genes (MSigDB). GSEA scores: mono/CC ES = 0.31 NES = 1.17, CC/Mono ES = −0.31, NES = −1.17, FDR = 0.487, *P* = 0.207.

As fibroblasts and mesenchymal cells often outgrow epithelial cells in cultures of primary mammary organoids from mouse or human, we assessed the growth properties of PHBECs and hAMSCs when co-cultured in the newly defined culture conditions. Equal numbers of tagged PHBECs and hAMSCs were co-cultured in suspension to enrich for precursors and generate heterotypic aggregates (see Additional file 5A). The aggregates were seeded onto the ECM-coated nylon substratum. Aggregates successfully attached to the substratum and formed locally constrained clusters that enlarged progressively. These clusters consisted of epithelial cells, mostly localized in the recesses of the meshes, covered by mesenchymal cells (Figure 2A, B). These results contrast with monocultures of PHBECs or hAMSCs, which invaded the substratum and covered larger areas (Figure 1D, Figure 3A). We concluded that hAMSCs do not outgrow PHBECs in these co-culture conditions but that each cell type constrains the growth and migration of the other.

### *Ex vivo* heterotypic culture conditions maintain mesenchymal and breast epithelial precursor cells

The cellular and molecular composition of the human breast environment that maintains undifferentiated human primary epithelial cells is as yet poorly defined. We tested whether epithelial and mesenchymal precursor cells in the co-cultures we developed might assemble an environment able to maintain undifferentiated breast cells in *ex vivo* long-term cultures.

We examined whether mature luminal cells are generated in *ex vivo* heterotypic cultures. GSEA analysis with specific signatures of co-culture and monoculture-derived epithelial cells demonstrated that epithelial cells maintained in *ex vivo* co-culture (CCE) are significantly enriched for luminal-specific genes, whereas epithelial cells maintained as monocultures (MCE) are not (see Additional file 5B). To further characterize the epithelial cells maintained in the *ex vivo* heterotypic culture conditions, we performed immunostaining and confocal microscopy of 40-day-old co-cultures. Immunostaining with EpCAM/K18 (see Additional file 5C), e-cadherin (CDH1, ECAD)/K18 (see Additional file 5D), and ERα/K18/K14 (see Additional file 5E) confirmed the presence of differentiated epithelial cells in heterotypic co-cultures. ERα staining was not detected in the co-cultures (see Additional file 5E). Further gene expression profiling of co-culture-derived epithelial cells showed that epithelial genes were among the most differentially over-expressed genes, whereas genes highly expressed in epithelial cells from monocultures were predominantly genes involved in the cell cycle (see Additional file 5F). FACS of dissociated cells from 70-day-old *ex vivo* co-cultures and corresponding monocultures stained with CD49f and EpCAM indicated the presence of different cell types in the co-cultures, with the EpCAM + CD49f + population being the most prominent (see Additional file 5G). We were not able to resolve these populations in the epithelial monocultures. These data further confirm that different breast cell populations are maintained in our long-term heterotypic co-cultures.

We then asked whether undifferentiated cells are also maintained in the *ex vivo* culture conditions described. Notably, immunostaining for the myoepithelial marker K14 or the luminal marker K19 and confocal microscopy of 60-day-old three-dimensional co-cultures of PHBECs and hAMSCs on ECM-coated meshes revealed the presence of K14/K19 double-positive cells, previously shown to be putative breast epithelial stem/progenitor cells [56]. K14/K19 cells were present at 1.5 ± 0.4% and located preferentially in the recesses of the substratum (Figure 2C-E). The remaining cells displayed either a myoepithelial (67 ± 10%) or a luminal (36.5 ± 9%) differentiation phenotype. Reconstitution of z-stacks of confocal frames showed that K14/K19 cells resided in close contact with differentiated epithelial cells (Figure 2D). These data suggest that breast epithelial and mesenchymal precursors grown on ECM-coated meshes generate an environment that maintains undifferentiated epithelial cells.

To address functionally the differentiation potential of cells growing in these structures, we dissociated 60-day-old *ex vivo* co-cultures and subjected the resulting single cells to colony formation assays. Of these cells, 1.7% ± 0.4% formed colonies: 1.1% ± 0.5% formed small colonies, whereas 0.6 ± 0.2% formed large colonies comprising luminal and myoepithelial cells (Figure 2F, G). Thus, the three-dimensional heterotypic culture system described here allows the assembly of structures that maintain bi-potent breast epithelial progenitor cells for 60 days *ex vivo*.

We also performed FACS of stromal cells from dissociated 60-day-old *ex vivo* co-cultures and assessed their differentiation potential. More than 60% of these cells differentiated into lipid-secreting or AP-positive cells in adipogenic or osteogenic culture conditions, respectively (see Additional file 5H), indicating that a population of mesenchymal precursor cells was maintained for 60 days *ex vivo*.

We subsequently investigated whether the epithelial cells in these heterotypic three-dimensional cultures can also differentiate into alveolar cells. When colonies arising from cells obtained from 30-day-old *ex vivo* co-cultures were exposed to lactogenic stimuli, they expressed the milk protein β-casein (Figure 2H). Taken together, our data demonstrate that tri-potent epithelial progenitor cells can be maintained in our *ex vivo* co-culture conditions.

### Transforming growth factor (TGF)β signaling is active in mesenchymal cells from co-cultures

Breast epithelial and mesenchymal precursors mutually constrain their growth and spreading in our *ex vivo* co-cultures (Figure 3A). Staining of 40-day-old *ex vivo* cultures for the proliferation marker Ki67 showed less proliferation in PHBECs and hAMSCs grown as co-cultures than as monocultures (Figure 3B). Consistently, GSEA analysis of the gene expression profiles of PHBECs or hAMSCs sorted by FACS revealed that PHBECs and hAMSCs grown as monocultures displayed significant expression of cell-cycle genes. In contrast, PHBECs and hAMSCs derived from co-cultures expressed neither cell-cycle genes (Figure 3C-E and see Additional files 6 and 7) nor apoptosis genes (Figure 3F, G).

**Figure 4:**
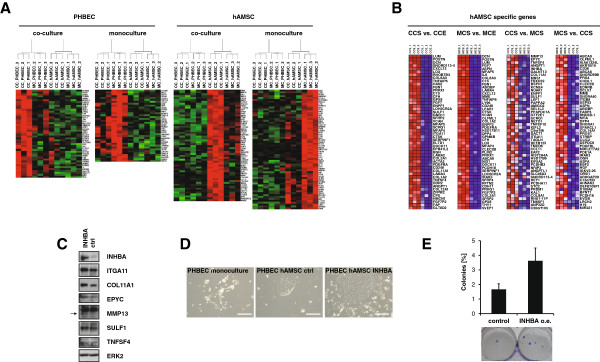
**Transforming growth factor (TGF)β signaling is active in mesenchymal cells from co-cultures and overexpression of inhibin β A (INHBA) in human adipose tissue-derived mesenchymal stem cells (hAMSCs) increases colony formation potential of epithelial cells. (A)** Heat maps derived from unsupervised hierarchical clustering of gene expression data of hAMSCs (n = 3) and primary human breast epithelial cells (PHBECs) (n = 3) grown as mono- or co-cultures for 21 to 30 days. Upregulated (red); downregulated (green); average expression (black). **(B)** Heat maps representing the top 50 upregulated genes specific for hAMSCs in monoculture (MCS) or co-cultures (CCS) compared with PHBECs in monocultures (MCE) or co-cultures (CCE) (left) and when compared among themselves (right). Upregulated (red); downregulated (blue); average expression (white). **(C)** Immunoblots with control and INHBA-overexpressing hAMSC lysates as indicated. Cells were grown on Primaria plates for 7 days. **(D)** Representative phase contrast pictures of colonies formed by single cells from 30-day-old PHBEC monocultures or PHBEC/hAMSC co-cultures. Co-cultures consisted of either control hAMSC or hAMSC overexpressing INHBA. Scale bar 50 μm. **(E)** Bar graph showing the percentage of colonies formed by single cells derived from *ex vivo* co-cultures of PHBECs and co-cultured with either control hAMSCs or hAMSCs overexpressing INHBA (INHBA o.e.). Representative pictures of crystal violet stained colonies (below).

Hierarchical clustering revealed distinct gene expression patterns of PHBECs and hAMSCs grown as co-cultures compared with those grown as monocultures (Figure 4A). Many genes were specifically downregulated in co-cultured compared with monocultured PHBECs (see Additional files 5F and 8, far right). Gene ontology mining and Ingenuity pathway analysis (IPA) also showed downregulation of genes involved in cell-cycle regulation (see Table 1 and Additional files 6 and 9A), further confirming that epithelial cells stay quiescent when co-cultured with mesenchymal cells.

**Table 1:** IPA upstream factor analysis of genes specifically expressed in PHBECs grown in co-cultures with hAMSCs

**Upstream regulator**	**Predicted activation state**	**Activation *z*-score**	***P*-value of overlap**
BNIP3L	Activated	3.05	5.60E-12
TP53	Activated	2.82	8.44E-12
let-7	Activated	2.01	1.12E-10
KDM5B	Activated	2.90	7.65E-07
CDKN2A	Activated	2.70	1.91E-06
MAPK14	Activated	2.28	5.65E-06
TCF3	Activated	3.32	1.49E-05
CEBPD	Activated	2.20	1.25E-04
PTHLH	Activated	2.20	1.47E-04
UXT	Activated	2.22	1.53E-04
TAZ	Activated	2.00	6.60E-04
MAP3K7	Activated	2.30	7.45E-04
SMARCB1	Activated	2.14	1.47E-03
P38 MAPK	Activated	2.29	6.01E-03
JUN	Activated	2.03	1.85E-02
Vegf	Inhibited	−2.35	1.70E-09
CCND1	Inhibited	−2.24	1.12E-08
MYC	Inhibited	−4.12	1.20E-08
E2F1	Inhibited	−2.89	2.51E-07
TBX2	Inhibited	−2.53	8.13E-07
FOXO1	Inhibited	−3.56	1.67E-05
MYBL2	Inhibited	−2.00	3.49E-03
MYCN	Inhibited	−2.38	7.17E-03
BMP7	Inhibited	−2.39	1.19E-02
CDKN1A		1.60	4.35E-21
HGF		−1.77	1.39E-10
CDK4			1.82E-09
TGFβ1		0.68	9.63E-08
RB1		1.14	8.23E-07
Tgf β		0.88	9.33E-06

PHBECs, primary human breast epithelial cells; hAMSCs: human adipose tissue-derived mesenchymal stem cells; IPA: Ingenuity pathway analysis.

Notably, changes in gene expression were more dramatic in co-cultured than in monocultured hAMSCs (Additional file 10 and Figure 4B right). ECM components, chemokines and cytokines were among the most significantly upregulated genes in co-cultured compared with monocultured hAMSCs (Figure 4B (right) and Additional file 10). IPA upstream factor analysis revealed TNF, IL1β, and TP53 to be inactive, whereas TGFβ1 was predicted to be active in hAMSCs grown in co-cultures (Table 2 and Additional file 9B). Downstream targets of several other components of TGFβ signaling were also significantly enriched among the genes specific for co-cultured hAMSC (Table 2).

**Table 2:** IPA upstream factor analysis of genes specifically expressed in hAMSCs grown in co-culture with PHBECs

**Upstream regulator**	**Predicted activation state**	**Activation z-score**	***P*-value of overlap**
TGFβ1	Activated	2,580	4,76E-16
SMAD2	Activated	2,204	7,91E-06
SMAD3	Activated	2,138	8,05E-06
NOTCH1	Activated	2,102	3,87E-05
Tgf β	Activated	2,241	5,70E-05
MKL1	Activated	2,744	4,28E-04
MKL2	Activated	2,449	2,49E-03
MGEA5	Activated	2,887	6,60E-03
INSIG1	Activated	2,621	9,33E-03
GFI1	Activated	2,236	1,56E-02
TNF	Inhibited	−2,756	1,52E-15
IL1B	Inhibited	−2,446	2,96E-09
TP53	Inhibited	−2,871	5,83E-08
NFkβ (complex)	Inhibited	−2,030	1,20E-07
CEBPA	Inhibited	−2,457	1,66E-06
TP63	Inhibited	−2,301	5,32E-06
RELA	Inhibited	−2,192	5,25E-05
PDGF BB	Inhibited	−2,883	2,02E-04
MYCN	Inhibited	−2,440	9,87E-04
ALDH2	Inhibited	−2,000	1,17E-03
CHUK	Inhibited	−2,101	1,39E-03
PPARG	Inhibited	−2,476	1,46E-03
TGFB3		1,286	4,60E-08
INHBA		−0.341	7.33E-05
TGFβR2		−0,296	1,59E-04
SMAD4		0,659	1,80E-04
BMP7		−0,464	2,17E-04

PHBECs, primary human breast epithelial cells; hAMSCs: human adipose tissue-derived mesenchymal stem cells; IPA: Ingenuity pathway analysis.

Inhibin β A *(INHBA)*, a member of the TGFβ family, was significantly upregulated in hAMSCs from co-cultures compared with monocultures (Figure 4B and Additional file 10). We asked whether INHBA contributed to the upregulation of other hAMSC co-culture signature genes (Figure 4B right and Additional file 10). Overexpression of INHBA in hAMSCs increased expression of integrin alpha 11 *(ITGA11)*, collagen type XI, alpha 1 *(COL11A1)*, epiphycan *(EPYC)*, and activated matrix metallopeptidase 13 *(MMP13)* but not the expression of sulfatase 1 *(SULF1)* or *TNFSF4* (Figure 4C). To assess the effects of INHBA overexpression in hAMSCs, we assembled cultures of PHBECs alone or with either control or INHBA-overexpressing hAMSCs (hAMSC INHBA), dissociated the cultures after 30 days, and subjected single cells to colony formation assays. Whereas epithelial cells from monocultures formed few colonies, epithelial cells derived from co-cultures of PHBECs and hAMSCs formed colonies at frequencies of 1.7 ± 0.4%. Notably, epithelial cells derived from co-cultures of PHBECs and hAMSC INHBA formed colonies at frequencies of 3.6 ± 0.9% (*P* = 0.023), indicating that constitutive overexpression of INHBA in hAMSCs increases the colony formation potential of PHBECs (Figure 4D, E).

## Discussion

Maintenance of the proliferation and differentiation potentials of non-immortalized primary human breast cells in long-term *ex vivo* cultures is of paramount importance for modeling and understanding heterotypic interactions in the normal breast. We have shown that primary epithelial and mesenchymal precursors isolated from normal human breast assemble heterotypic structures that allow long-term maintenance of primary breast progenitor cells, which are able to differentiate into luminal and myoepithelial cells, and into cells that produce a milk protein upon exposure to lactogenic cues.

We have used a cell culture system consisting of M5 medium, oxygen at 5% and an ECM-coated substratum. A defined serum-free medium was used because serum composition varies from lot to lot, contains undefined components, and influences differentiation [57]. Free radicals generated by non-physiological oxygen tensions (approximately 20%) in standard cultures harm primary cells and compromise their proliferation and differentiation [52,53,67,68]. Moreover, recent evidence indicates that low oxygen tensions are crucial for the maintenance of mesenchymal and hematopoietic stem cells [69,70]. In addition to a low oxygen tension in the incubators, the M5 medium was enriched with antioxidants to further protect cells from reactive oxygen species [68]. The ECM-coated substratum provides a scaffold for three-dimensional growth of primary cells, while allowing nutrient and growth factor diffusion. These conditions allowed long-term heterotypic *ex vivo* culture of primary breast cells, maintaining undifferentiated epithelial and mesenchymal precursors as well as differentiated luminal and myoepithelial cells.

Recently, a further elegant study has used human primary cells and a three-dimensional scaffold to reconstitute a multicellular culture model of human breast tissue [71]. We reconstituted long-term heterotypic co-cultures based on freshly isolated differentiation-competent breast mesenchymal and stromal precursor cells in serum-free, oxygen-controlled cell culture conditions with a rigid ECM-coated scaffold. In contrast, Wang *et al*. made use of more differentiated human mammary epithelial cells (HuMECs, Invitrogen) and human mammary fibroblasts (HMF, ScienCell) as well as human adipose-derived stem cells (hASCs, Jeffrey Gimble, Pennington Biomedical Research Center, LA, USA) in silk protein scaffolds and serum-containing cell culture medium at 20% oxygen tension. Whereas we aimed to dissect mechanisms involved in stromal epithelial reciprocity by characterizing changes in stromal and epithelial cells upon heterotypic co-culture compared to monoculture, Wang *et al*. assessed response to hormone stimulation in their multicellular culture system. Hence both studies describe highly valuable experimental model systems based on human primary cells that recapitulate the complexity of human tissue *ex vivo*.

It has been proposed that stem/progenitor niche structures include various cell types and secreted ECM components [15,17,72,73]. This concept has been proposed in rodent and human mammary tissue [8,17,18,56,72] but the exact characteristics and components of the niche remain ill-defined. Mesenchymal cells were shown to contribute to stem cell niches in the hematopoietic system and the gastrointestinal tract [20,22,74]. Here we provide evidence that heterotypic cultures of breast primary epithelial and mesenchymal precursors on coated meshes maintain their proliferation and differentiation potentials. We further show that co-cultures of hAMSCs and PHBECs dramatically increase TGFβ signaling in mesenchymal cells and that INHBA release by mesenchymal cells in heterotypic co-cultures increases the colony formation capacity of epithelial cells.

The heterotypic cultures of human breast cells described here will allow further molecular characterization of non-immortalized stromal and epithelial cells, and thereby, identify critical signaling circuits maintaining the differentiation and proliferation potentials of breast epithelial and mesenchymal precursors. This is particularly important given the involvement of aberrant differentiation and of deregulated stroma-epithelial crosstalk in tumorigenesis. In addition, these heterotypic three-dimensional cultures, together with the possibility to transduce primary breast epithelial cells with up to five different transgenes [41], offers a framework for testing the relevance of potential oncogenic events identified by high-throughput sequencing of breast cancer genomes [31-33]. Moreover, given that different subtypes of breast cancer are thought to arise from cells with distinct differentiation states [2,3], the culture conditions described here should allow development of pathophysiologically relevant *ex vivo* breast cancer models to test the ever-increasing number of targeted therapies in development.

## Conclusions

Our results demonstrate that heterotypic culture of breast primary epithelial and mesenchymal precursors on coated meshes maintains their proliferation and differentiation potential and constrains their growth reciprocally. Further, we provide evidence that mesenchymal INHBA increases the colony formation potential of epithelial cells. This co-culture system will prove useful for further characterization of the molecular mechanisms mediating interactions between human normal or neoplastic breast epithelial cells and the stroma.

## Abbreviations

AP: alkaline phosphatase; COL11A1: collagen type XI, alpha 1; DAPI: 4',6-diamidino-2-phenylindole, DMEM, Dulbecco's modified Eagle's medium; DsRed2: red fluorescent protein; ECM: extracellular Matrix; eGFP: enhanced green fluorescent protein; EPYC: epiphycan; ES: enrichment score; FACS: fluorescence-activated cell sorting; FDR: false discovery rate; GSEA: gene set enrichment analysis; hAMSCs: human adipose tissue-derived mesenchymal stem cells; HEPES: 4-(2-hydroxyethyl)-1-piperazineethanesulfonic acid; IL1β: interleukin 1β; INHBA: inhibin β A; ITGA11: integrin alpha 11; Ki67: Ki-67 proliferation marker; Lin-: lineage-negative; MMP13: matrix metallopeptidase 13 (collagenase 3); MSC: mesenchymal stem cells; NES: normalized enrichment score; PBS: phosphate-buffered saline; PHBECs: primary human breast epithelial cells; SULF1: sulfatase 1; TGFβ1: transforming growth factor, β 1; TNF: tumor necrosis factor; ULA: ultra-low attachment.

## Competing interests

Some information in this publication is related to a patent application (patent number WO 2012/143407 (26.10.12), Culture medium suitable for the culture of undifferentiated cells) by The Friedrich Miescher Institute for Biomedical Research. MBA and SD are listed as inventors on this application. All other authors declare no competing interests.

## Authors’ contributions

SD conceived the study, designed the experiments, collected, assembled and analyzed the data, interpreted the results, and wrote the manuscript. HB contributed to primary breast tissue processing and organoid preparation, analyzed the data, interpreted the results and revised the manuscript. AB performed immunofluorescence staining and statistical analysis, and also revised the manuscript. EC performed RNA extractions and processed small cell number microarrays, analyzed the data, and revised the manuscript; DMF and DJS participated in the conception of the study, provided breast reduction mammoplasty tissue for the study, assured informed patient consent, and revised the manuscript; MBA conceived the study, designed the experiments, interpreted the results, wrote the manuscript, and provided financial support. All authors read and approved the final manuscript.

## Supplementary Material

Additional file 1 **Flow cytometry sorting strategy for the isolation of different epithelial and mesenchymal cell populations from freshly dissociated breast tissue organoids. (A)** Gate to exclude cell debris is shown. **(B)** Gate to exclude duplets. **(C)** Gate to exclude lineage positive cells. **(D)** Gate to exclude dead cells. **(E)** Gates to sort EpCAM + and CD49+ EpCAM- epithelial cells plus gate to separate mesenchymal cells. **(F)** Gates to sort CD10+ and CD10- mesenchymal cells. Click here for file

Additional file 2 **(A) M5 medium maintains the differentiation and proliferation potentials of primary luminal epithelial cells.** Representative images of K18- (red) and K14- (green) immunofluorescent staining of primary human breast epithelial cells (PHBECs) cultured in MEGM, HMM or M5 medium. Bar graph showing the percentage area covered by cells (left) and the percentage K18-positive cells (right). *P <0.01, **P <0.16, ***P <0.002, ****P <0.0002. Scale bars 100 μm. **(B)** Luminal and myoepithelial cells when cultured in M5 medium are smaller than in HMM or MEGM medium. Bar graph (upper) shows the average size of luminal cells (K18 positive) after 28 days culture in MEGM, HMM or M5 medium. *P = 4.1E-5, **P = 0.04. Bar graph (lower) shows the average size of myoepithelial cells (K14 positive) after 28 days culture in MEGM, HMM or M5 medium. *P = 1.7E-8, **P = 0.002. Cell size was measured with ImageJ [75] software; 30 to 100 cells from three different experiments were analyzed. **(C)** Representative image of mammospheres grown in M5 medium and a bar graph showing mammosphere formation frequencies in M5, MEGM and HMM medium. **(D)** Representative immunofluorescent staining of PHBEC colonies after colony formation assays in M5 medium; antibodies p63 (red), K18 (blue) and K14 (green). Scale bar 100 μm. **(E)** Human adipose tissue-derived mesenchymal stem cells (hAMSCs) can be differentiated into cells expressing adipocyte or osteocyte markers. Representative images of Oil Red O staining marking lipids in adipocytes (upper) or osteocyte-specific alkaline phosphatase (AP) staining (lower). Control conditions: mesenchymal stem cell medium (MSCM); differentiation conditions: MSCM medium supplemented with adipogenic (upper) or osteogenic differentiation factors (lower). Scale bars 50 μm. **(F)** Human breast mesenchymal and epithelial cells can be infected and tagged with lentiviruses expressing fluorescent marker proteins. Representative phase-contrast (upper) and fluorescence (lower) images of PHBECs tagged with VenusNLS and of hAMSCs expressing red fluorescent protein (DsRed2). Scale bars 50 μm. Click here for file

Additional file 3 **Mesenchymal- and epithelial-specific genes group human adipose tissue-derived mesenchymal stem cell (hAMSC) or primary human breast epithelial cell (PHBEC) samples, respectively. (A)** Pearson correlation heat map matrix of gene expression profiles from three epithelial and three mesenchymal monocultures grown for 30 days in *ex vivo* culture conditions. **(B)** Heat map with clusters derived from unsupervised hierarchical clustering of gene expression data of hAMSCs (n = 3) and PHBECs (n = 3) grown as monocultures on an ECM-coated mesh for 21 to 30 days. Gene cluster enriched for up regulated mesenchymal genes (left); gene cluster enriched for upregulated epithelial genes (right). Upregulated (red); downregulated (green); average expression (black). Click here for file

Additional file 4 **(A, B) Mesenchymal and epithelial genes are enriched in human adipose tissue-derived mesenchymal stem cells (hAMSCs) and primary human breast epithelial cells (PHBECs), respectively.** Heat maps representing the top 50 gene set enrichment analysis (GSEA)-ranked genes specific for monocultures of hAMSCs (left) (n = 3) and monocultures of PHBECs (right) (n = 3). Upregulated (red); downregulated (blue); average (*white*). **(C, D)** PHBECs preserve their epithelial phenotype and display molecular characteristics of luminal progenitor cells when cultured in *ex vivo* conditions on extracellular matrix (ECM)-coated meshes for 30 days. **(C)** GSEA with PHBEC-specific genes and signatures of EpCAM + epithelial cells and CD10+ myoepithelial and myofibroblast cells [63]. PHBEC/EpCAM+: Enrichment score (ES) = 0.56, normalized enrichment score (NES) = 1.96, false discovery rate (FDR) <0.001, *P* <0.001; PHBEC/CD10+: ES = −0.76, NES = −2.54, FDR <0.001, *P* < 0.001 **(D)**. GSEA with PHBEC-specific genes and signatures of EpCAM + and CD49f + luminal progenitor cells and a EpCAM − and CD49f + basal cell population [64]. PHBEC/EpCAM + CD49f+: ES = 0.66, NES = 2.11, FDR <0.001, *P* <0.001; PHBEC/EpCAM − CD49f+: ES = −0.56, NES = −2.29, FDR <0.001, *P* <0.001 **(E)**. GSEA with PHBEC-specific genes and signatures of BPEC or HMEC, respectively [1]. PHBEC/BPEC: ES = 0.56, NES = 1.53, FDR = 0.01, *P* = 0.006; PHBEC/HMEC: ES = −0.45, NES = −1.33, FDR = 0.1, *P* = 0.09. **(F, G)** hAMSCs cultured in *ex vivo* conditions maintain mesenchymal stem cell (MSC) gene expression profiles. GSEA with hAMSC or PHBEC signatures and MSC signatures. For Pedemonte *et al*. [66]: hAMSC: ES = 0.7, NES = 1.38, FDR = 0.13, *P* <0.001; PHBEC: ES = −0.7, NES = −1.4, FDR = 0.097, *P* < 0.001. For Huang *et al*. [65]: hAMSC: ES = 0.82, NES = 1.31, FDR = 0.092, *P* <0.001; PHBEC: ES = −0.82, NES = −1.3, FDR = 1.116, *P* <0.001. Click here for file

Additional file 5 **(A) Phase-contrast (upper left) and fluorescence images of mixed aggregates from primary human breast epithelial cells (PHBECs) expressing enhanced green fluorescent protein (EGFP) and h human adipose tissue-derived mesenchymal stem cells (hAMSCs) expressing red fluorescent protein (DsRed2) maintained in suspension.** Scale bar 300 μm. **(B)** Epithelial cells maintained as co-cultures (CCE) are significantly enriched in luminal genes, whereas epithelial cells maintained in monocultures (SCE) are not. CCE and MCE-specific genes were identified by comparison (gene set enrichment analysis (GSEA) analysis of variance (ANOVA)) to each other or to co-culture stromal cells (CCS). The signatures were compared (GSEA) to signatures of EpCAM + CD49f − luminal cells [64]. CCE versus CCS: enrichment score (ES) = 0.45, normalized enrichment score (NES) = 1.58, false discovery rate (FDR) <0.005, *P* <0.005; CCE versus MCE: ES = 0.43, NES = 1.80, FDR <0.005, *P* <0.005; MCE versus CCS: ES = 0.29, NES = 0.99, FDR = 0.47, *P* = 0.47. **(C-E)** Confocal images of EpCAM/K18 **(C)**, ECAD (CDH1)/K18 **(D)**, and K14/K18/ERα stained 40-day-old co-cultures of PHBECs and hAMSCs grown on an extracellular matrix (ECM)-coated mesh: 4',6-diamidino-2-phenylindole (DAPI)- (blue) stained nuclei. Scale bars 40 μm. **(F)** Epithelial genes are upregulated in PHBECs from co-cultures (CCE). Heat maps representing the top 50 upregulated genes specific for PHBECs in monoculture (MCE) or co-cultures (CCE) compared among themselves (right) or with hAMSCs in monocultures (MCS) or co-cultures (CCS) (left). Upregulated (red); downregulated (blue); average (*white*). **(G)** Fluorsence-actived cell sorting (FACS) plots of dissociated cells from 70-day-old co-culture (CC), PHBEC and hAMSC monocultures (MCE, MCS). Cells were stained with CD49f-FITC, EpCAM-PerCP Cy5.5 and DAPI. Only viable cells (DAPI−) were analyzed. **(H)** Mesenchymal precursor cells are maintained in long-term co-cultures. Images of Oil-Red-O staining marking lipids in adipocytes (upper) or osteocyte-specific alkaline phosphatase (AP) staining (lower) after differentiation of FACS-sorted mesenchymal precursor cells from 60-day-old co-cultures. Control: M5 medium; differentiation conditions: M5 medium supplemented with adipogenic (upper) or osteogenic differentiation factors (lower). Scale bars 100 μm. Click here for file

Additional file 6 **Genes involved in cell cycle and cell division are significantly upregulated in monocultured primary human breast epithelial cells (PHBECs), whereas co-cultured PHBECs do not express these genes.** Gene Ontology (GO) mining was performed with significantly upregulated genes in monocultured PHBECs compared to co-cultured PHBECs. The parent–child union algorithm with Bonferroni correction (*P*-value (Adj)) was applied in ontologizer [48]. The most significant GO terms are shown. Click here for file

Additional file 7 **Genes involved in cell cycle progression and DNA replication are downregulated in co-cultured primary human breast epithelial cells (PHBECs).** Gene set enrichment analysis (GSEA) was performed with genes that are upregulated in monocultured PHBECs and downregulated in co-cultured PHBECs. Most significant Molecular Signatures Database (MSigDB) gene sets (GS) enriched in monocultured PHBECs are shown. ES, enrichment score; NES, normalized enrichment score; NOM *P-value*, Nominal *P*-value, statistical significance of the enrichment score; FDR, false discovery rate (<0.25 is significant). Click here for file

Additional file 8 **Most significantly upregulated and downregulated genes in primary human breast epithelial cells (PHBECs) in *ex vivo *co-cultures.** LogFC_CCE-MCE, computed log fold change of comparison of gene expression levels in PHBECs in co-cultures (CCE) versus PHBECs in monocultures (MCE) (see Material and methods). pVal_CCE-MCE, *P*-value representing significance of comparison. Click here for file

Additional file 9 **Primary human breast epithelial cells (PHBECs) in heterotypic *ex vivo *cultures are quiescent and human adipose tissue-derived mesenchymal stem cells (hAMSCs) show activated transforming growth factor (TGF)β1 signaling. (A)** Downstream regulated targets of CCND1 and p16 (CDKN2A) in co-culture PHBECs displayed as networks. **(B)** Downstream regulated targets of TGFβ1 in co-culture hAMSCs displayed as networks. Orange shapes, active regulators; blue shapes, inactive regulators; red-shaded shapes, upregulated targets; green-shaded shapes, downregulated targets; gray lines, activity not predictable; yellow lines, activity contradictive. Click here for file

Additional file 10 **Most significantly upregulated and downregulated genes in human adipose tissue-derived mesenchymal stem cells (hAMSCs) in *ex vivo *co-cultures.** LogFC_CCS-MCS, computed log-fold change in comparison of gene expression levels in hAMSCs in co-cultures (CCS) versus hAMSCs in monocultures (MCS) (see Material and methods). pVal_CCS-MCS, *P*-value representing significance of comparison. Click here for file
