# Supplementary material for: Mesenchymal precursor cells maintain the differentiation and proliferation potentials of breast epithelial cells
Source: Breast Cancer Res. 2014 Jun 10;16(3):R60. doi: 10.1186/bcr3673 (PMC4095576; doi:10.1186/bcr3673)
Supplement: Additional file 1 — Flow cytometry sorting strategy for the isolation of different epithelial and mesenchymal cell populations from freshly dissociated breast tissue organoids. (A) Gate to exclude cell debris is shown. (B) Gate to exclude duplets. (C) Gate to exclude lineage positive cells. (D) Gate to exclude dead cells. (E) Gates to sort EpCAM + and CD49+ EpCAM- epithelial cells plus gate to separate mesenchymal cells. (F) Gates to sort CD10+ and CD10- mesenchymal cells. [file bcr3673-S1.pdf]

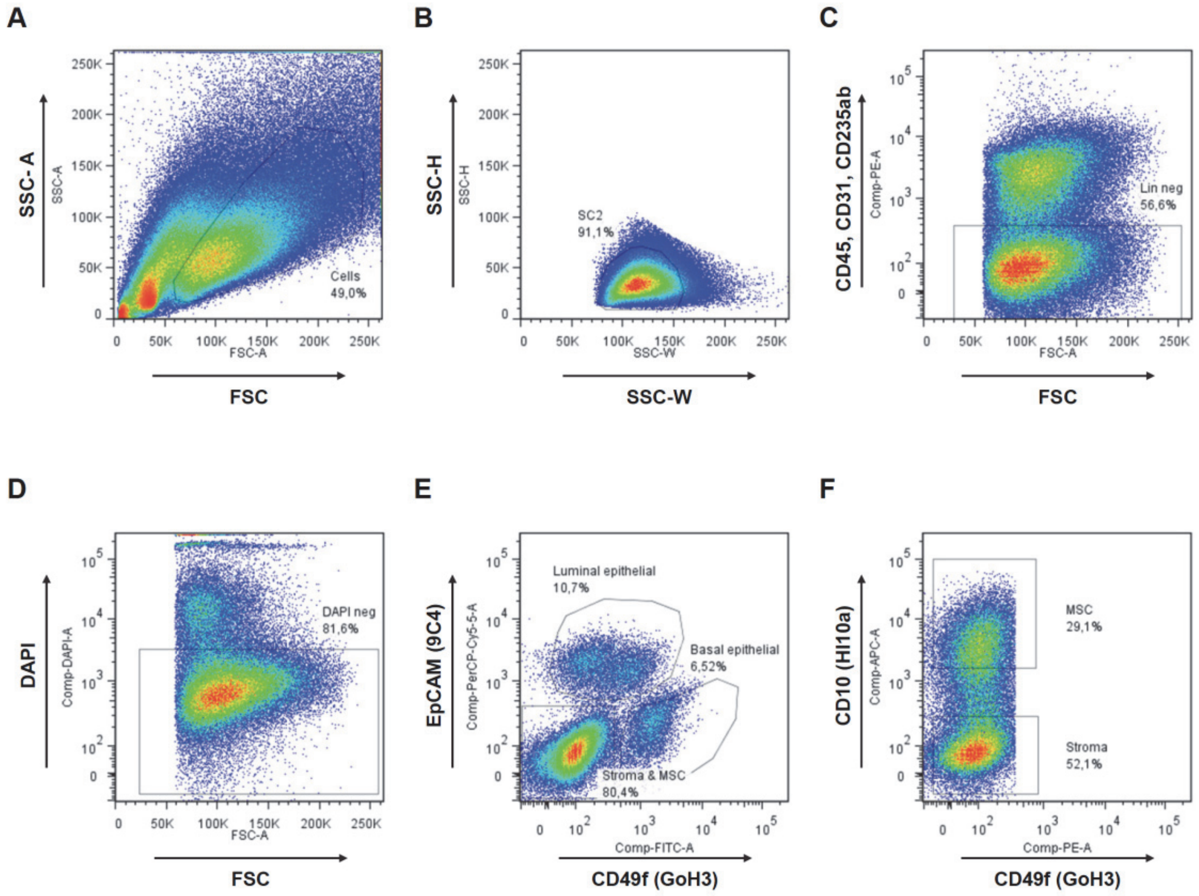

Additional file 1: Flow cytometry sorting strategy for the isolation of different epithelial and mesenchymal cell populations from freshly dissociated breast tissue organoids. (A) Gate to exclude cell debris is shown. (B) Gate to exclude duplets. (C) Gate to exclude lineage positive Cells. (D) Gate to exclude dead cells. (E) Gates to sort EpCAM+ and CD49+ EpCAM- epithelial cells plus gate to separate mesenchymal cells. (F) Gates to sort CD10+ and CD10- mesenchymal cells.
