# Supplementary figures and images for: Mesenchymal precursor cells maintain the differentiation and proliferation potentials of breast epithelial cells
Source: Breast Cancer Res. 2014 Jun 10;16(3):R60. doi: 10.1186/bcr3673 (PMC4095576; doi:10.1186/bcr3673)

Additional file 2

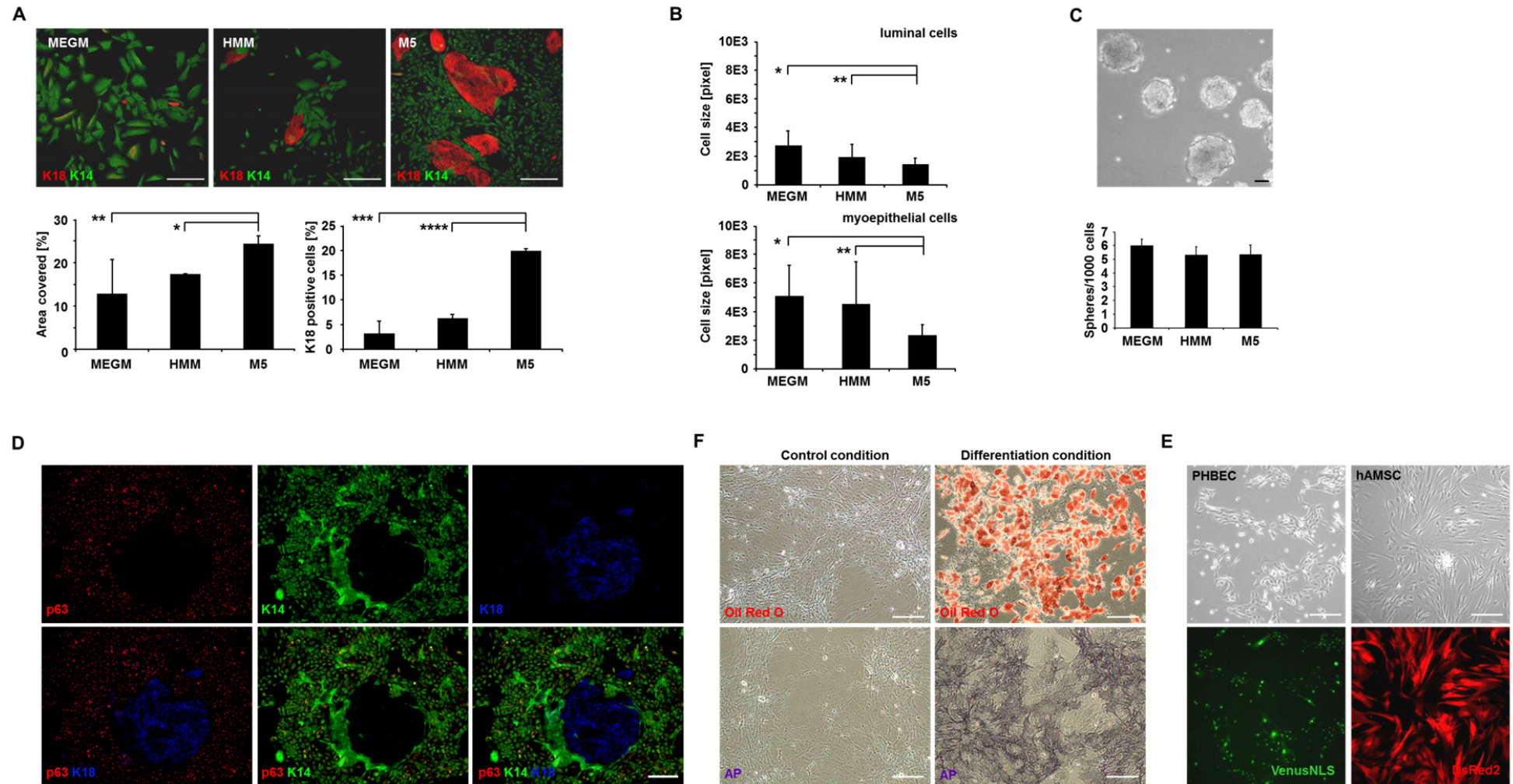

Supplement: Additional file 2 — (A) M5 medium maintains the differentiation and proliferation potentials of primary luminal epithelial cells. Representative images of K18- (red) and K14- (green) immunofluorescent staining of primary human breast epithelial cells (PHBECs) cultured in MEGM, HMM or M5 medium. Bar graph showing the percentage area covered by cells (left) and the percentage K18-positive cells (right). *P <0.01, **P <0.16, ***P <0.002, ****P <0.0002. Scale bars 100 μm. (B) Luminal and myoepithelial cells when cultured in M5 medium are smaller than in HMM or MEGM medium. Bar graph (upper) shows the average size of luminal cells (K18 positive) after 28 days culture in MEGM, HMM or M5 medium. *P = 4.1E-5, **P = 0.04. Bar graph (lower) shows the average size of myoepithelial cells (K14 positive) after 28 days culture in MEGM, HMM or M5 medium. *P = 1.7E-8, **P = 0.002. Cell size was measured with ImageJ [75] software; 30 to 100 cells from three different experiments were analyzed. (C) Representative image of mammospheres grown in M5 medium and a bar graph showing mammosphere formation frequencies in M5, MEGM and HMM medium. (D) Representative immunofluorescent staining of PHBEC colonies after colony formation assays in M5 medium; antibodies p63 (red), K18 (blue) and K14 (green). Scale bar 100 μm. (E) Human adipose tissue-derived mesenchymal stem cells (hAMSCs) can be differentiated into cells expressing adipocyte or osteocyte markers. Representative images of Oil Red O staining marking lipids in adipocytes (upper) or osteocyte-specific alkaline phosphatase (AP) staining (lower). Control conditions: mesenchymal stem cell medium (MSCM); differentiation conditions: MSCM medium supplemented with adipogenic (upper) or osteogenic differentiation factors (lower). Scale bars 50 μm. (F) Human breast mesenchymal and epithelial cells can be infected and tagged with lentiviruses expressing fluorescent marker proteins. Representative phase-contrast (upper) and fluorescence (lower) images of PHBECs tagge [file bcr3673-S2.pdf]

## Additional file 5

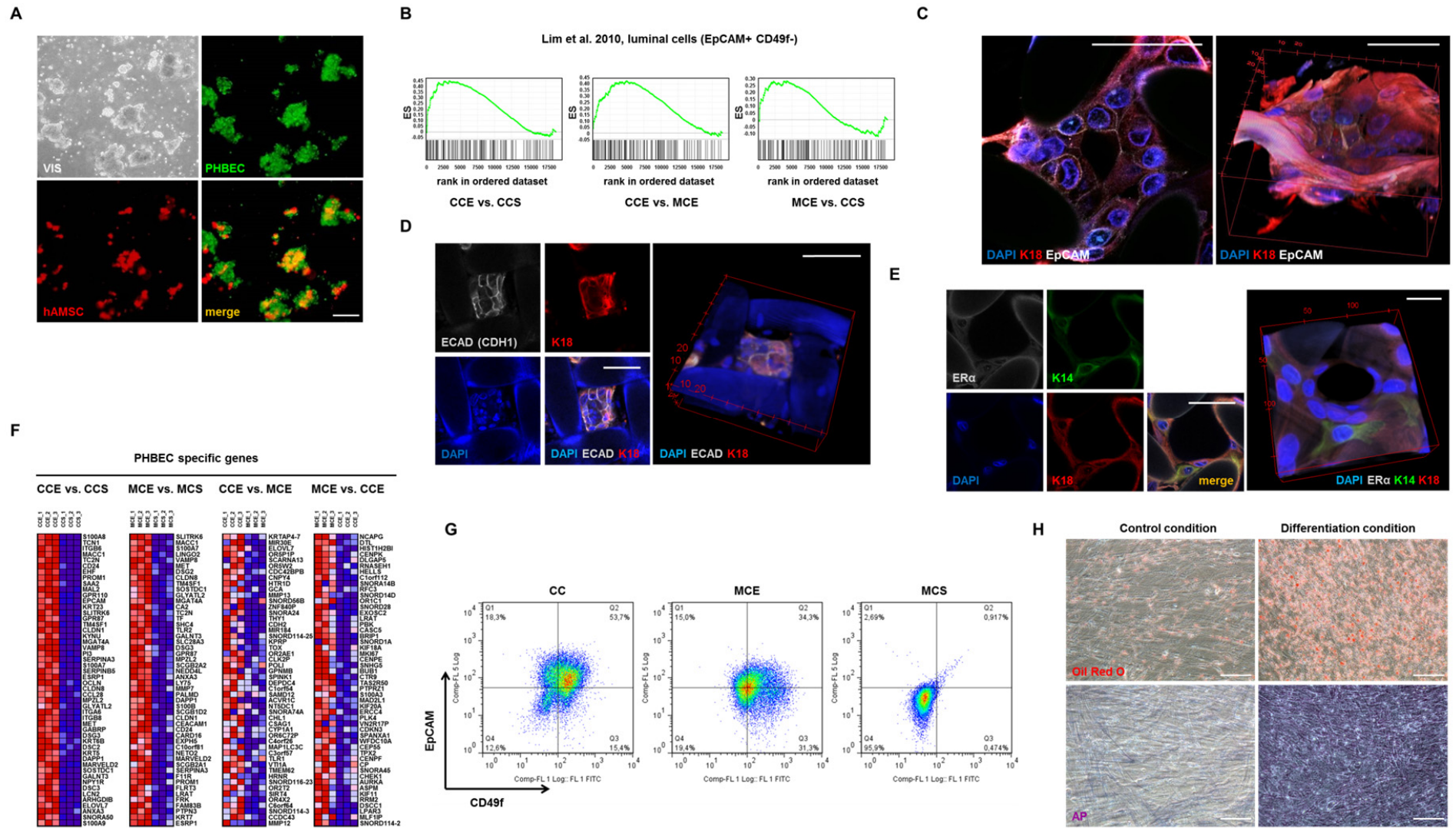

Supplement: Additional file 5 — (A) Phase-contrast (upper left) and fluorescence images of mixed aggregates from primary human breast epithelial cells (PHBECs) expressing enhanced green fluorescent protein (EGFP) and h human adipose tissue-derived mesenchymal stem cells (hAMSCs) expressing red fluorescent protein (DsRed2) maintained in suspension. Scale bar 300 μm. (B) Epithelial cells maintained as co-cultures (CCE) are significantly enriched in luminal genes, whereas epithelial cells maintained in monocultures (SCE) are not. CCE and MCE-specific genes were identified by comparison (gene set enrichment analysis (GSEA) analysis of variance (ANOVA)) to each other or to co-culture stromal cells (CCS). The signatures were compared (GSEA) to signatures of EpCAM + CD49f − luminal cells [64]. CCE versus CCS: enrichment score (ES) = 0.45, normalized enrichment score (NES) = 1.58, false discovery rate (FDR) <0.005, P <0.005; CCE versus MCE: ES = 0.43, NES = 1.80, FDR <0.005, P <0.005; MCE versus CCS: ES = 0.29, NES = 0.99, FDR = 0.47, P = 0.47. (C-E) Confocal images of EpCAM/K18 (C), ECAD (CDH1)/K18 (D), and K14/K18/ERα stained 40-day-old co-cultures of PHBECs and hAMSCs grown on an extracellular matrix (ECM)-coated mesh: 4',6-diamidino-2-phenylindole (DAPI)- (blue) stained nuclei. Scale bars 40 μm. (F) Epithelial genes are upregulated in PHBECs from co-cultures (CCE). Heat maps representing the top 50 upregulated genes specific for PHBECs in monoculture (MCE) or co-cultures (CCE) compared among themselves (right) or with hAMSCs in monocultures (MCS) or co-cultures (CCS) (left). Upregulated (red); downregulated (blue); average (white). (G) Fluorsence-actived cell sorting (FACS) plots of dissociated cells from 70-day-old co-culture (CC), PHBEC and hAMSC monocultures (MCE, MCS). Cells were stained with CD49f-FITC, EpCAM-PerCP Cy5.5 and DAPI. Only viable cells (DAPI−) were analyzed. (H) Mesenchymal precursor cells are maintained in long-term co-cultures. Images of Oil-Red-O staining marking lipids in adipocy [file bcr3673-S5.pdf]
