# Supplementary material for: Mesenchymal precursor cells maintain the differentiation and proliferation potentials of breast epithelial cells
Source: Breast Cancer Res. 2014 Jun 10;16(3):R60. doi: 10.1186/bcr3673 (PMC4095576; doi:10.1186/bcr3673)
Supplement: Additional file 3 — Mesenchymal- and epithelial-specific genes group human adipose tissue-derived mesenchymal stem cell (hAMSC) or primary human breast epithelial cell (PHBEC) samples, respectively. (A) Pearson correlation heat map matrix of gene expression profiles from three epithelial and three mesenchymal monocultures grown for 30 days in ex vivo culture conditions. (B) Heat map with clusters derived from unsupervised hierarchical clustering of gene expression data of hAMSCs (n = 3) and PHBECs (n = 3) grown as monocultures on an ECM-coated mesh for 21 to 30 days. Gene cluster enriched for up regulated mesenchymal genes (left); gene cluster enriched for upregulated epithelial genes (right). Upregulated (red); downregulated (green); average expression (black). [file bcr3673-S3.pdf]

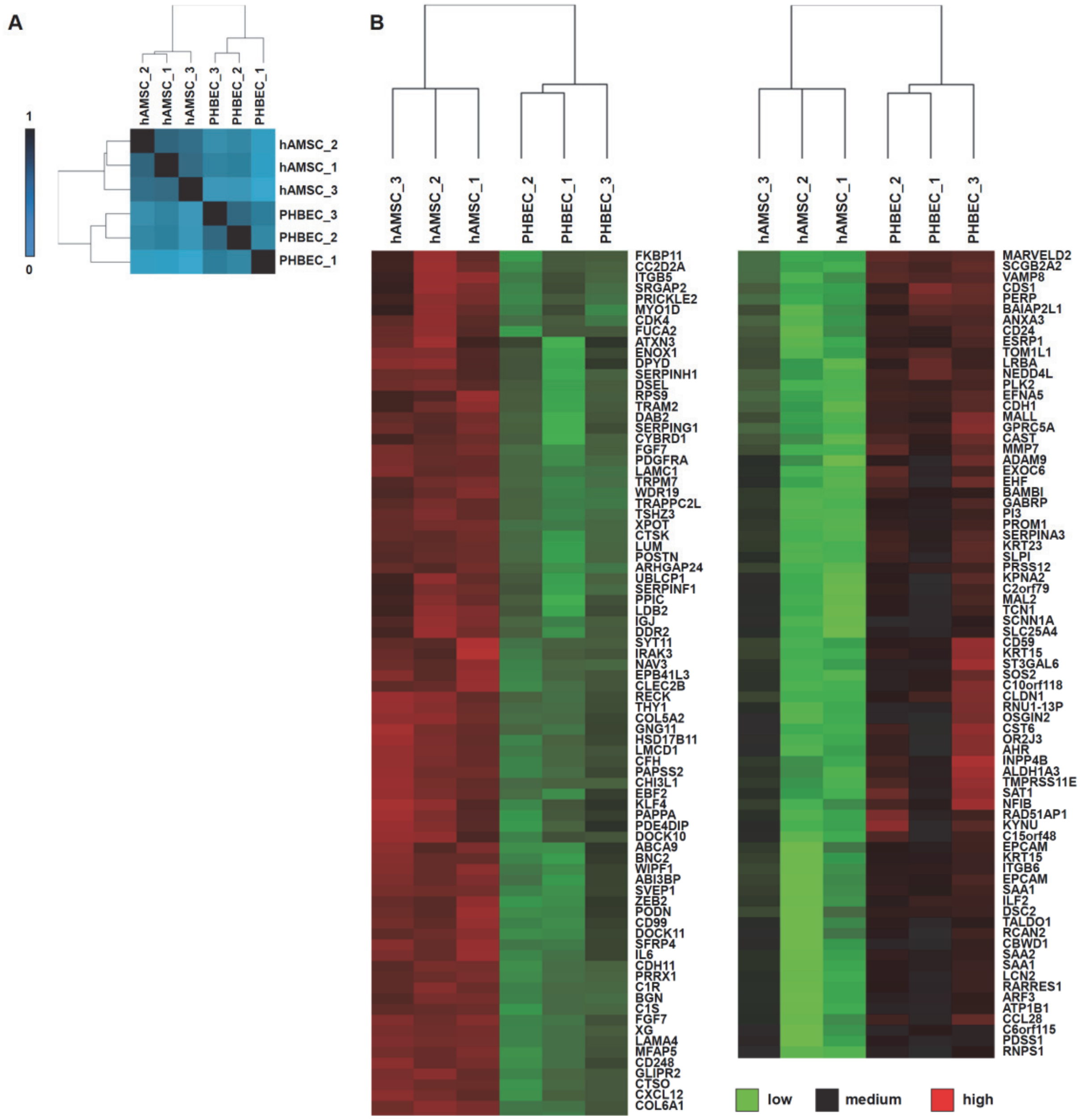

Additional file 3: Mesenchymal- and epithelial-specific genes group hAMSC or PHBEC samples, respectively. (A) Pearson correlation heat map matrix of the gene expression profiles from three epithelial and three mesenchymal monocultures grown for 30 days in *ex vivo* culture conditions. **(B)** Heat map with clusters derived from unsupervised hierarchical clustering of gene expression data of hAMSCs (n=3) and PHBECs (n=3) grown as monocultures on an ECM-coated mesh for 21-30 days. (*Left*) Gene-cluster enriched for up regulated mesenchymal genes. (*Right*) Gene cluster enriched for upregulated epithelial genes. (*Red*) up regulated; (*Green*) downregulated; (*Black*) average expression.
