# Supplementary material for: Mesenchymal precursor cells maintain the differentiation and proliferation potentials of breast epithelial cells
Source: Breast Cancer Res. 2014 Jun 10;16(3):R60. doi: 10.1186/bcr3673 (PMC4095576; doi:10.1186/bcr3673)
Supplement: Additional file 4 — (A, B) Mesenchymal and epithelial genes are enriched in human adipose tissue-derived mesenchymal stem cells (hAMSCs) and primary human breast epithelial cells (PHBECs), respectively. Heat maps representing the top 50 gene set enrichment analysis (GSEA)-ranked genes specific for monocultures of hAMSCs (left) (n = 3) and monocultures of PHBECs (right) (n = 3). Upregulated (red); downregulated (blue); average (white). (C, D) PHBECs preserve their epithelial phenotype and display molecular characteristics of luminal progenitor cells when cultured in ex vivo conditions on extracellular matrix (ECM)-coated meshes for 30 days. (C) GSEA with PHBEC-specific genes and signatures of EpCAM + epithelial cells and CD10+ myoepithelial and myofibroblast cells [63]. PHBEC/EpCAM+: Enrichment score (ES) = 0.56, normalized enrichment score (NES) = 1.96, false discovery rate (FDR) <0.001, P <0.001; PHBEC/CD10+: ES = −0.76, NES = −2.54, FDR <0.001, P < 0.001 (D). GSEA with PHBEC-specific genes and signatures of EpCAM + and CD49f + luminal progenitor cells and a EpCAM − and CD49f + basal cell population [64]. PHBEC/EpCAM + CD49f+: ES = 0.66, NES = 2.11, FDR <0.001, P <0.001; PHBEC/EpCAM − CD49f+: ES = −0.56, NES = −2.29, FDR <0.001, P <0.001 (E). GSEA with PHBEC-specific genes and signatures of BPEC or HMEC, respectively [1]. PHBEC/BPEC: ES = 0.56, NES = 1.53, FDR = 0.01, P = 0.006; PHBEC/HMEC: ES = −0.45, NES = −1.33, FDR = 0.1, P = 0.09. (F, G) hAMSCs cultured in ex vivo conditions maintain mesenchymal stem cell (MSC) gene expression profiles. GSEA with hAMSC or PHBEC signatures and MSC signatures. For Pedemonte et al. [66]: hAMSC: ES = 0.7, NES = 1.38, FDR = 0.13, P <0.001; PHBEC: ES = −0.7, NES = −1.4, FDR = 0.097, P < 0.001. For Huang et al. [65]: hAMSC: ES = 0.82, NES = 1.31, FDR = 0.092, P <0.001; PHBEC: ES = −0.82, NES = −1.3, FDR = 1.116, P <0.001. [file bcr3673-S4.pdf]

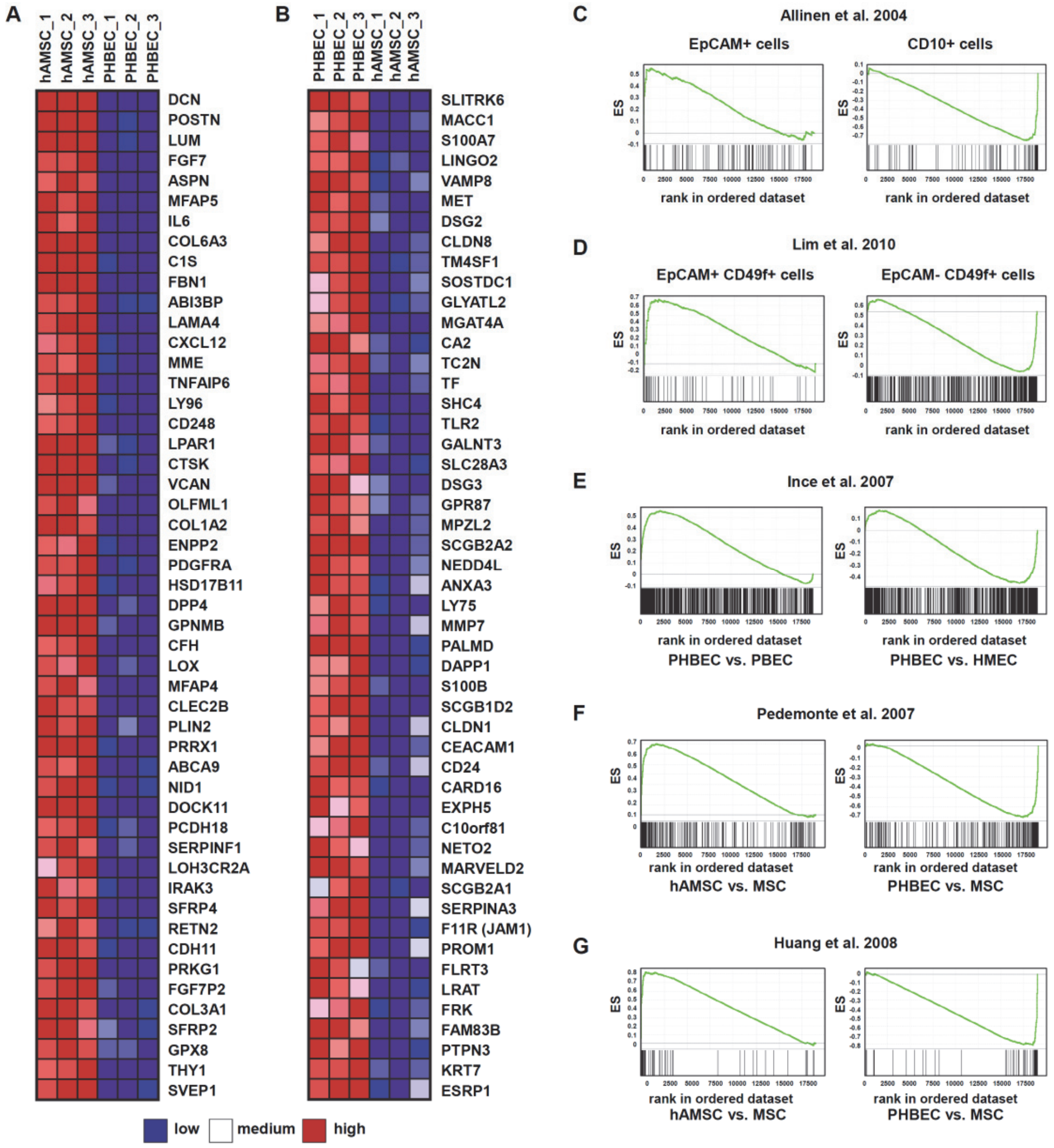


**Additional file 4: (A, B) GSEA-ranked lists reveal that mesenchymal and epithelial genes are enriched in hAMSCs and PHBECs respectively.** Heat maps representing the top 50 GSEA-ranked genes specific for monocultures of hAMSCs (Left) (n=3) and monocultures of PHBECs (Right) (n=3). (Red) upregulated; (Blue) downregulated; (White) average expression. **(C, D) PHBECs preserve their epithelial phenotype and display molecular characteristics of luminal progenitor cells when cultured in *ex vivo* conditions on ECM-coated meshes for 30 days. (C)** GSEA with PHBEC-specific genes and published signatures of EpCAM positive epithelial cells and CD10 positive myoepithelial and myofibroblast cells (Allinen et al., 2004). PHBEC/EpCAM+: ES=0.56, NES=1.96, FDR<0.001, *P*<0.001; PHBEC/CD10+: ES=-0.76, NES=-2.54, FDR<0.001, *P*<0.001 (D) GSEA with PHBEC-specific genes and published signatures of EpCAM positive and CD49f positive luminal progenitor cells and EpCAM

negative and a CD49f positive basal cell population (Lim et al., 2010). PHBEC/EpCAM+ CD49f+: ES=0.66, NES=2.11, FDR<0.001, *P*>0.001; PHBEC/EpCAM- CD49f+: ES=-0.56, NES=-2.29, FDR<0.001, *P*>0.001 **(E)** GSEA with PHBEC-specific genes and published signatures of "BPEC" or "HMEC", respectively 1. PHBEC/BPEC: ES=0.56, NES=1.53, FDR=0.01, P=0.006; PHBEC/HMEC: ES=-0.45, NES=-1.33, FDR=0.1, P=0.09. **(F, G) hAMSCs cultured in *ex vivo* conditions maintain mesenchymal stem cell gene expression profiles.** GSEA with hAMSC or PHBEC signatures and published MSC signatures. For Pedemonte et al.60: hAMSC: ES=0.7, NES=1.38, FDR=0.13, P<0.001; PHBEC: ES=-0.7, NES=-1.4, FDR=0.097, P<0.001. For Huang et al.59: hAMSC: ES= 0.82, NES=1.31, FDR=0.092, P<0.001; PHBEC: ES=-0.82, NES=-1.3, FDR=1.116, P<0.001. ES enrichment score, NES normalized enrichment score, FDR false discovery rate.
