# Supplementary material for: Mesenchymal precursor cells maintain the differentiation and proliferation potentials of breast epithelial cells
Source: Breast Cancer Res. 2014 Jun 10;16(3):R60. doi: 10.1186/bcr3673 (PMC4095576; doi:10.1186/bcr3673)
Supplement: Additional file 6 — Genes involved in cell cycle and cell division are significantly upregulated in monocultured primary human breast epithelial cells (PHBECs), whereas co-cultured PHBECs do not express these genes. Gene Ontology (GO) mining was performed with significantly upregulated genes in monocultured PHBECs compared to co-cultured PHBECs. The parent–child union algorithm with Bonferroni correction (P-value (Adj)) was applied in ontologizer [48]. The most significant GO terms are shown. [file bcr3673-S6.pdf]

Additional file 6: Genes involved in cell cycle and cell division are significantly upregulated in monocultured PHBECs whereas co-cultured PHBECs do not express these genes. Gene Ontology mining was performed with significantly up-regulated genes in monocultured PHBECs when compared to co-cultured PHBECs. The parent child union algorithm with Bonferroni correction (p-Value (Adj)) was applied in ontologizer (http://compbio.charite.de/ontologizer/). The most significant GO terms are shown.

ID	Name	p-Value	p-Value (Adj)	size
GO:0022402	cell cycle process	1.19E-20	1.83E-17	44/690
GO:0007049	cell cycle	1.96E-17	3.02E-14	47/960
GO:0051301	cell division	3.55E-16	5.47E-13	28/332
GO:0007059	chromosome segregation	3.74E-14	5.76E-11	16/97
GO:0006260	DNA replication	2.92E-11	4.50E-08	18/226
GO:0051726	regulation of cell cycle	3.61E-11	5.56E-08	26/443
GO:0048285	organelle fission	7.85E-11	1.21E-07	28/273
GO:0006259	DNA metabolic process	4.11E-08	6.32E-05	25/566
GO:0000819	sister chromatid segregation	9.52E-08	0.000146	11/36
