# Supplementary material for: Mesenchymal precursor cells maintain the differentiation and proliferation potentials of breast epithelial cells
Source: Breast Cancer Res. 2014 Jun 10;16(3):R60. doi: 10.1186/bcr3673 (PMC4095576; doi:10.1186/bcr3673)
Supplement: Additional file 7 — Genes involved in cell cycle progression and DNA replication are downregulated in co-cultured primary human breast epithelial cells (PHBECs). Gene set enrichment analysis (GSEA) was performed with genes that are upregulated in monocultured PHBECs and downregulated in co-cultured PHBECs. Most significant Molecular Signatures Database (MSigDB) gene sets (GS) enriched in monocultured PHBECs are shown. ES, enrichment score; NES, normalized enrichment score; NOM P-value, Nominal P-value, statistical significance of the enrichment score; FDR, false discovery rate (<0.25 is significant). [file bcr3673-S7.pdf]

Additional file 7: Genes involved in cell cycle progression and DNA replication are downregulated in co-cultured PHBECs. Gene set enrichment analysis (GSEA) was performed with genes that are upregulated in monocultured PHBECs and downregulated in co-cultured PHBECs. Most significant Molecular Signatures Database (MSigDB) gene sets (GS) enriched in mono-cultured PHBECs are shown. ES, enrichment score; NES, normalized enrichment score; NOM p-val, Nominal p value, statistical significance of the enrichment score; FDR, false discovery rate (<0.25 is significant).

**GS MSigDB**	**ES**	**NES**	**NOM p-val**	**FDR q-val**
KEGG_CELL_CYCLE	0.58	2.35	0.000	0.000
KEGG_DNA_REPLICATION	0.71	2.34	0.000	0.000
KEGG_MISMATCH_REPAIR	0.72	2.12	0.000	0.000
KEGG_PURINE_METABOLISM	0.47	2.03	0.000	0.002
KEGG_NITROGEN_METABOLISM	0.68	2.02	0.000	0.002
KEGG_SPLICEOSOME	0.49	2.00	0.000	0.003
KEGG_RNA_DEGRADATION	0.55	1.98	0.000	0.003

KEGG_PYRIMIDINE_METABOLISM	0.49	1.97	0.000	0.003
KEGG_HOMOLOGOUS_RECOMBINATION	0.64	1.95	0.000	0.003
KEGG_CITRATE_CYCLE_TCA_CYCLE	0.60	1.90	0.002	0.006
