# Supplementary material for: Mesenchymal precursor cells maintain the differentiation and proliferation potentials of breast epithelial cells
Source: Breast Cancer Res. 2014 Jun 10;16(3):R60. doi: 10.1186/bcr3673 (PMC4095576; doi:10.1186/bcr3673)
Supplement: Additional file 8 — Most significantly upregulated and downregulated genes in primary human breast epithelial cells (PHBECs) in ex vivo co-cultures. LogFC_CCE-MCE, computed log fold change of comparison of gene expression levels in PHBECs in co-cultures (CCE) versus PHBECs in monocultures (MCE) (see Material and methods). pVal_CCE-MCE, P-value representing significance of comparison. [file bcr3673-S8.pdf]

Additional file 8: Most significantly upregulated and downregulated genes in PHBECs in *ex vivo* co-cultures.

Gene_ID	logFC_CCE-MCE	pVal_CCE-MCE	Description
ELOVL7	2.035469	0.001263	ELOVL family member 7, elongation of long chain fatty acids (yeast)
LOC100131541	2.020525	0.00028	hypothetical LOC100131541
MIR34A	1.47331	0.001561	microRNA 34a
DTX2	1.466542	0.000477	deltex homolog 2 (Drosophila)
MIR30E	1.437259	0.004363	microRNA 30e
LY6G5B	1.376098	0.003527	lymphocyte antigen 6 complex, locus G5B
DAB2	1.205069	0.000163	disabled homolog 2, mitogen-responsive phosphoprotein (Drosophila)
BCL2L1	1.112348	0.002831	BCL2-like 1 (BCLX, BCL-XL)
HTR1D	1.090916	0.003013	5-hydroxytryptamine (serotonin) receptor 1D
CLK2P	1.066583	0.002706	CDC-like kinase 2, pseudogene
C6orf64	1.045358	0.000967	chromosome 6 open reading frame 64
SNRPN	-4.17541	0.000525	small nuclear ribonucleoprotein polypeptide N
SNORD50A	-3.37755	0.000603	small nucleolar RNA, C/D box 50A
NCAPG	-2.57053	0.001031	non-SMC condensin I complex, subunit G

SNORD38B	-2.44372	0.004579	small nucleolar RNA, C/D box 38B
DTL	-2.32172	0.000162	denticleless homolog (Drosophila)
RNASEH1	-2.22394	0.003523	ribonuclease H1
HIST1H2BI	-1.82815	0.000601	histone cluster 1, H2bi
LRAT	-1.81516	0.002081	lecithin retinol acyltransferase (phosphatidylcholine--retinol O-acyltransferase)
CTR9	-1.78041	0.000584	Ctr9, Paf1/RNA polymerase II complex component, homolog (S. cerevisiae)
LRRC37A2	-1.53067	0.002527	leucine rich repeat containing 37, member A2
SPANXA2	-1.50638	0.000466	SPANX family, member A2
HELLS	-1.38695	0.004072	helicase, lymphoid-specific
POLD3	-1.36429	0.00155	polymerase (DNA-directed), delta 3, accessory subunit
CA5B	-1.35463	0.001753	carbonic anhydrase VB, mitochondrial
KIAA1586	-1.33864	0.001957	KIAA1586
SCYL3	-1.33287	0.001241	SCY1-like 3 (S. cerevisiae)
ERCC4	-1.29095	0.002365	excision repair cross-complementing rodent repair deficiency, complementation group 4
BRIP1	-1.25474	1.18E-05	BRCA1 interacting protein C-terminal helicase 1
LSM6	-1.23915	0.002825	LSM6 homolog, U6 small nuclear RNA associated (S. cerevisiae)
GLTSCR2	-1.22346	0.001803	glioma tumor suppressor candidate region gene 2

HMGB2	-1.18979	0.002779	high-mobility group box 2
TIMP1	-1.16642	0.000645	TIMP metallopeptidase inhibitor 1
COPZ2	-1.16295	0.003243	coatomer protein complex, subunit zeta 2
XPO6	-1.15291	0.000856	exportin 6
ZFYVE16	-1.14447	2.09E-05	zinc finger, FYVE domain containing 16
PTPRZ1	-1.13278	0.001864	protein tyrosine phosphatase, receptor-type, Z polypeptide 1
KCTD14	-1.09396	0.001772	potassium channel tetramerisation domain containing 14
KIAA1598	-1.08906	0.004336	KIAA1598
DPYD	-1.07097	0.001397	dihydropyrimidine dehydrogenase
C21orf91	-1.05873	0.002727	chromosome 21 open reading frame 91
POGK	-1.05455	0.001731	pogo transposable element with KRAB domain
TAF2	-1.04992	0.000708	TAF2 RNA polymerase II, TATA box binding protein (TBP)-associated factor, 150kDa
ZNF562	-1.02308	0.000299	zinc finger protein 562
LBR	-1.02224	0.001805	lamin B receptor
CDC7	-1.01102	0.004452	cell division cycle 7 homolog (S. cerevisiae)
CALML4	-1.00426	0.003622	calmodulin-like 4
CCDC41	-1.0008	0.001017	coiled-coil domain containing 41
