# Supplementary material for: Mesenchymal precursor cells maintain the differentiation and proliferation potentials of breast epithelial cells
Source: Breast Cancer Res. 2014 Jun 10;16(3):R60. doi: 10.1186/bcr3673 (PMC4095576; doi:10.1186/bcr3673)
Supplement: Additional file 9 — Primary human breast epithelial cells (PHBECs) in heterotypic ex vivo cultures are quiescent and human adipose tissue-derived mesenchymal stem cells (hAMSCs) show activated transforming growth factor (TGF)β1 signaling. (A) Downstream regulated targets of CCND1 and p16 (CDKN2A) in co-culture PHBECs displayed as networks. (B) Downstream regulated targets of TGFβ1 in co-culture hAMSCs displayed as networks. Orange shapes, active regulators; blue shapes, inactive regulators; red-shaded shapes, upregulated targets; green-shaded shapes, downregulated targets; gray lines, activity not predictable; yellow lines, activity contradictive. [file bcr3673-S9.pdf]

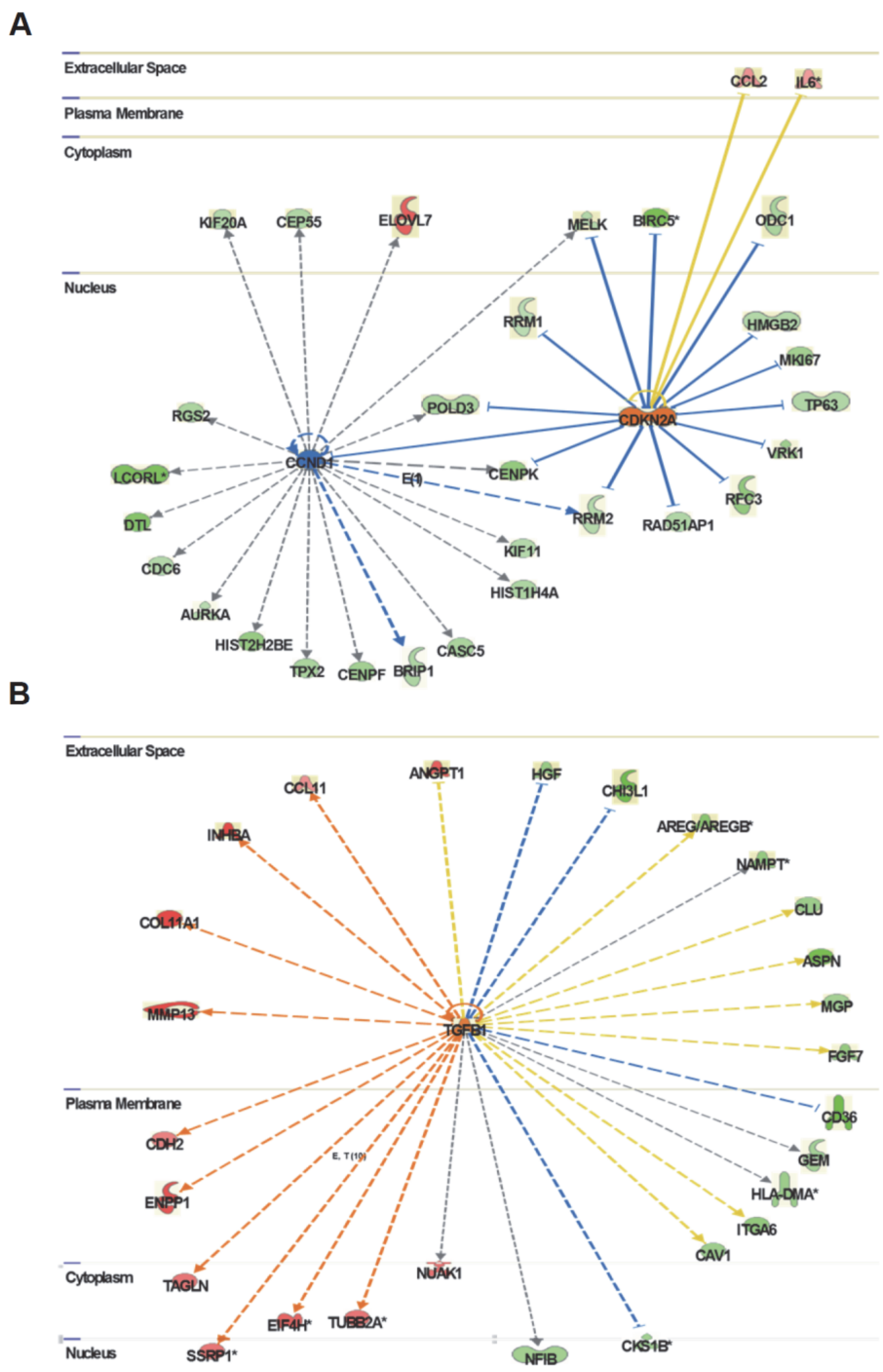

Additional file 9: PHBECs in heterotypic *ex vivo* cultures are quiescent and hAMSCs show activated TGFß1 signaling. (A) Downstream regulated targets of CCND1 and p16 (CDKN2A) in co-culture PHBECs displayed as networks. **(B)** Downstream regulated targets of TGFß1 in co-culture hAMSCs displayed as networks. Orange shapes: active regulators, blue shapes: inactive regulators, Red shaded shapes: upregulated targets, Green shaded shapes: downregulated targets, grey lines: activity not predictable, yellow lines: activity contradictive.
