# Supplementary material for: Mesenchymal precursor cells maintain the differentiation and proliferation potentials of breast epithelial cells
Source: Breast Cancer Res. 2014 Jun 10;16(3):R60. doi: 10.1186/bcr3673 (PMC4095576; doi:10.1186/bcr3673)
Supplement: Additional file 10 — Most significantly upregulated and downregulated genes in human adipose tissue-derived mesenchymal stem cells (hAMSCs) in ex vivo co-cultures. LogFC_CCS-MCS, computed log-fold change in comparison of gene expression levels in hAMSCs in co-cultures (CCS) versus hAMSCs in monocultures (MCS) (see Material and methods). pVal_CCS-MCS, P-value representing significance of comparison. [file bcr3673-S10.pdf]

Additional file 10: Most significantly upregulated and downregulated genes in hAMSCs in *ex vivo* co-cultures

**Gene_ID**	**logFC_CCS-MCS**	**pVal_CCS-MCS**	**Description**
MMP13	4.640723	0.000125	matrix metallopeptidase 13 (collagenase 3)
INHBA	3.091423	0.001351	inhibin, beta A
COL11A1	2.929676	9.45E-09	collagen, type XI, alpha 1
LOC401097	2.892429	8.73E-06	Similar to LOC166075
ENPP1	2.656819	8.06E-05	ectonucleotide pyrophosphatase/phosphodiesterase 1
SULF1	2.552532	0.000158	sulfatase 1
ANGPT1	2.460727	7.78E-06	angiopoietin 1
FBXO32	2.344134	0.001208	F-box protein 32
EPYC	2.319811	0.0001	epiphycan
TNFSF4	2.278077	0.000125	tumor necrosis factor (ligand) superfamily, member 4
ITGA11	2.131321	1.40E-06	integrin, alpha 11
NUAK1	2.041484	9.88E-08	NUAK family, SNF1-like kinase, 1
GALNT13	2.039363	0.000797	UDP-N-acetyl-alpha-D-galactosamine:polypeptide N-acetylgalactosaminyltransferase 13
DACT1	1.845281	0.000225	dapper, antagonist of beta-catenin, homolog 1 (Xenopus laevis)

MNS1	1.658473	0.000297	meiosis-specific nuclear structural 1
CCL11	1.556619	9.44E-06	chemokine (C-C motif) ligand 11
SLC38A1	1.530776	0.001437	solute carrier family 38, member 1
TPM1	1.391483	1.81E-05	tropomyosin 1 (alpha)
FLJ33996	1.391243	0.000293	hypothetical protein FLJ33996
PAPPA2	1.369824	0.001278	pappalysin 2
COMP	1.203057	0.000349	cartilage oligomeric matrix protein
MRAS	1.190434	0.000203	muscle RAS oncogene homolog
ANTXR1	1.142667	0.000533	anthrax toxin receptor 1
SNORA70	1.128091	0.001007	small nucleolar RNA, H/ACA box 70
COL8A1	1.11093	0.001962	collagen, type VIII, alpha 1
BGN	1.107134	0.000473	biglycan
PDLIM3	1.084033	0.000701	PDZ and LIM domain 3
TSNAX	1.081397	0.000861	translin-associated factor X
KIAA1199	1.066886	6.02E-05	KIAA1199
PALLD	1.02927	0.000918	palladin, cytoskeletal associated protein
COL1A1	1.005134	0.001674	collagen, type I, alpha 1

UCK2	1.000584	0.00031	uridine-cytidine kinase 2
DPP4	-3.8413	0.000506	dipeptidyl-peptidase 4
CHI3L1	-3.64973	0.000116	chitinase 3-like 1 (cartilage glycoprotein-39)
ABI3BP	-3.45556	2.59E-05	ABI family, member 3 (NESH) binding protein
CD36	-3.17878	0.001115	CD36 molecule (thrombospondin receptor)
MME	-3.12409	3.73E-05	membrane metallo-endopeptidase
ABCA9	-2.98074	2.32E-07	ATP-binding cassette, sub-family A (ABC1), member 9
ASPN	-2.91795	0.000167	asporin
FABP3	-2.73031	8.75E-05	fatty acid binding protein 3, muscle and heart (mammary-derived growth inhibitor)
USP53	-2.59178	0.000156	ubiquitin specific peptidase 53
PRG4	-2.55087	0.000712	proteoglycan 4
KIAA1324L	-2.48533	0.000125	KIAA1324-like
H19	-2.48121	6.98E-06	H19, imprinted maternally expressed transcript (non-protein coding)
OLFML1	-2.42899	6.87E-06	olfactomedin-like 1
COL14A1	-2.28444	8.97E-05	collagen, type XIV, alpha 1
DEPDC6	-2.26407	0.000138	DEP domain containing 6
CLEC2B	-2.25898	2.14E-06	C-type lectin domain family 2, member B

ITGB8	-2.11175	0.000523	integrin, beta 8
NFIA	-2.04056	8.87E-05	nuclear factor I/A
IFT80	-2.03646	0.000832	intraflagellar transport 80 homolog (Chlamydomonas)
DCLK1	-2.03198	2.13E-05	doublecortin-like kinase 1
PCDH18	-2.02456	0.000935	protocadherin 18
KCNK2	-1.96443	0.000856	potassium channel, subfamily K, member 2
EBF2	-1.95366	1.46E-06	early B-cell factor 2
AHNAK2	-1.9387	0.0005	AHNAK nucleoprotein 2
PLIN2	-1.93471	0.000484	perilipin 2
EVI2B	-1.89637	0.001493	ecotropic viral integration site 2B
EDNRB	-1.87448	6.69E-05	endothelin receptor type B
LPAR1	-1.86136	5.53E-05	lysophosphatidic acid receptor 1
EFEMP1	-1.86048	0.000674	EGF-containing fibulin-like extracellular matrix protein 1
STRBP	-1.71553	0.001013	spermatid perinuclear RNA binding protein
HLA-DMA	-1.7072	0.00117	major histocompatibility complex, class II, DM alpha
PDGFRL	-1.69995	0.001927	platelet-derived growth factor receptor-like
LAMA4	-1.62257	0.000905	laminin, alpha 4

OR5I1	-1.61237	0.001527	olfactory receptor, family 5, subfamily I, member 1
SLC43A3	-1.60662	0.000124	solute carrier family 43, member 3
CREB5	-1.51225	0.00031	cAMP responsive element binding protein 5
GEM	-1.50438	0.000747	GTP binding protein overexpressed in skeletal muscle
C14orf43	-1.49981	0.000791	chromosome 14 open reading frame 43
IRAK3	-1.47566	0.001532	interleukin-1 receptor-associated kinase 3
OGN	-1.4656	5.31E-06	osteoglycin
MIRLET7A2	-1.35017	4.41E-07	microRNA let-7a-2
AIM1	-1.32869	0.000301	absent in melanoma 1
SPARCL1	-1.32237	5.27E-05	SPARC-like 1 (hevin)
RFX8	-1.31985	0.000103	regulatory factor X, 8
TTC19	-1.30115	0.001601	tetratricopeptide repeat domain 19
HMG20A	-1.2917	0.001041	high-mobility group 20A
DDB1	-1.28993	0.000961	damage-specific DNA binding protein 1, 127kDa
KHDRBS3	-1.23053	0.001647	KH domain containing, RNA binding, signal transduction associated 3
FDFT1	-1.18182	0.00035	farnesyl-diphosphate farnesyltransferase 1
RND3	-1.1498	0.000939	Rho family GTPase 3

OSBPL1A	-1.13985	0.001997	oxysterol binding protein-like 1A
ADSL	-1.09503	0.000132	adenylosuccinate lyase
USP24	-1.06974	0.000333	ubiquitin specific peptidase 24
NFKBIA	-1.02545	0.0015	nuclear factor of kappa light polypeptide gene enhancer in B-cells inhibitor, alpha
SNX9	-1.02365	2.96E-05	sorting nexin 9
PDE1A	-1.00186	8.62E-07	phosphodiesterase 1A, calmodulin-dependent
